# Rotating Magnetic Field Increases β-Lactam Antibiotic Susceptibility of Methicillin-Resistant *Staphylococcus aureus* Strains

**DOI:** 10.3390/ijms222212397

**Published:** 2021-11-17

**Authors:** Marta Woroszyło, Daria Ciecholewska-Juśko, Adam Junka, Radosław Drozd, Marcin Wardach, Paweł Migdał, Patrycja Szymczyk-Ziółkowska, Daniel Styburski, Karol Fijałkowski

**Affiliations:** 1 Department of Microbiology and Biotechnology, Faculty of Biotechnology and Animal Husbandry, West Pomeranian University of Technology in Szczecin, Piastów 45, 70-311 Szczecin, Poland; marta.woroszylo@zut.edu.pl (M.W.); daria.ciecholewska@zut.edu.pl (D.C.-J.); radoslaw.drozd@zut.edu.pl (R.D.); 2 Department of Pharmaceutical Microbiology and Parasitology, Faculty of Pharmacy, Medical University of Wroclaw, Borowska 211a, 50-534 Wrocław, Poland; 3 Laboratory of Microbiology, Łukasiewicz Research Network–PORT Polish Center for Technology Development, 54-066 Wrocław, Poland; 4 Faculty of Electrical Engineering, West Pomeranian University of Technology in Szczecin, Sikorskiego 37, 70-313 Szczecin, Poland; marcin.wardach@zut.edu.pl; 5 Department of Environment, Hygiene and Animal Welfare, Faculty of Biology and Animal Science, Wroclaw University of Environmental and Life Sciences, Chełmońskiego 38C, 51-630 Wrocław, Poland; pawel.migdal@upwr.edu.pl; 6 Centre for Advanced Manufacturing Technologies (CAMT/FPC), Faculty of Mechanical Engineering, Wroclaw University of Science and Technology, Łukasiewicza 5, 50-371 Wrocław, Poland; patrycja.e.szymczyk@pwr.edu.pl; 7 Laboratory of Chromatography and Mass Spectroscopy, Faculty of Biotechnology and Animal Husbandry, West Pomeranian University of Technology in Szczecin, Klemensa Janickiego 29, 71-270 Szczecin, Poland; daniel.styburski@zut.edu.pl

**Keywords:** antibiotics, antibiotic resistance, MRSA, *Staphylococcus aureus*, rotating magnetic field

## Abstract

Methicillin-resistant strains of *Staphylococcus aureus* (MRSA) have developed resistance to most β-lactam antibiotics and have become a global health issue. In this work, we analyzed the impact of a rotating magnetic field (RMF) of well-defined and strictly controlled characteristics coupled with β-lactam antibiotics against a total of 28 methicillin-resistant and sensitive *S. aureus* strains. The results indicate that the application of RMF combined with β-lactam antibiotics correlated with favorable changes in growth inhibition zones or in minimal inhibitory concentrations of the antibiotics compared to controls unexposed to RMF. Fluorescence microscopy indicated a drop in the relative number of cells with intact cell walls after exposure to RMF. These findings were additionally supported by the use of SEM and TEM microscopy, which revealed morphological alterations of RMF-exposed cells manifested by change of shape, drop in cell wall density and cytoplasm condensation. The obtained results indicate that the originally limited impact of β-lactam antibiotics in MRSA is boosted by the disturbances caused by RMF in the bacterial cell walls. Taking into account the high clinical need for new therapeutic options, effective against MRSA, the data presented in this study have high developmental potential and could serve as a basis for new treatment options for MRSA infections.

## 1. Introduction

In recent years, antimicrobial resistance has become a major public health issue. Methicillin-resistant strains of *Staphylococcus aureus* (referred to as MRSA) have developed resistance to most β-lactam antibiotics including penicillins, cephalosporins (with the exception of ceftaroline and ceftobiprole) and carbapenems [1]. The frequency of occurrence of these resistant strains (compared to methicillin-sensitive *S. aureus* strains, referred to as MSSA) has been reported for 25% and, in certain geographical regions, even over 50% of all SA strains. The Centers for Disease Control and Prevention estimates that MRSA is responsible for more than 70,000 severe infections and 9000 deaths [2]. MRSA has been identified as the most common etiological factor of skin and soft tissue infections in United States intensive care hospital units and it is also associated with numerous other disease entities, including biomaterial-related diseases [3,4]. It is believed that even ongoing development of new (other than β-lactam) antibiotics and advances in infection prevention will not prevent MRSA from remaining one of the most prominent pathogens with persistently high mortality [5]. The main reason behind the above-mentioned evolutionary success of this pathogen in the nosocomial and, in recent years, community environments, is the fact that MRSA harbors the *mecA* gene which codes alternative penicillin-binding protein, PBP2a, responsible for the resistance mechanism in question. Although other gene variants, namely *mecB* and *mecC,* occur less frequently in the staphylococcal genome, their presence is also related with resistance to β-lactams [6,7]. The native PBP protein is the essential enzyme in the process of bacterial cell wall synthesis. β-lactam antibiotics, against which MRSA strains are resistant, exert their antibacterial activity by binding and inactivating PBPs. Importantly, β-lactam antibiotics display a low affinity for alternative PBP2a, so the process of cell wall synthesis mediated by this protein remains undisturbed. This results in the survival of MRSA strains in the presence of β-lactam antibiotics [8]. Currently, numerous alternative strategies have been developed to overcome the issues related to microbial resistance [9]. Among them, the application of various types of magnetic fields (MFs) has been proposed to boost the activity of standard antimicrobial agents [10,11,12,13]. 

The first studies on the influence of magnetic fields (MFs) on organisms started at the end of the 19th century. This line of investigation was intensified in subsequent decades, mostly fueled by a growing interest in the impact of fields generated by electric and telecommunication networks on the health and behavior of humans and such animals as bees and birds [14]. At present, it is well-established that MFs may affect structure and functional processes in microorganisms. Regarding the direct effects of MFs on microorganisms, the literature indicates possible modifications in the physiology and shape of the cells, changes in the chemical-physical characteristics of the cellular membrane [15], and in membrane permeability [16,17]. The most commonly observed exposure effects concern changes in growth dynamics [18,19,20,21], the ability of bacteria to adhere and form biofilms [22,23], gene transcription [24,25] and sensitivity to antimicrobial substances [26], as well as irreversible damage to the microorganisms, mostly due to the loss of integrity of the cellular wall/membrane [27,28,29,30,31]. 

Despite the existence of seemingly extensive scientific literature on the topic, the results of studies of the influence of various types of MFs on microorganisms are not unequivocal. Some authors reported an antibacterial effect of MFs [32,33,34,35], while others suggested the lack of any significant impact of MFs on microbial growth [36,37], biochemical activity [38] or bacterial adhesion [39]. Other research teams demonstrated a stimulating effect of MFs on microbial cell growth and cell viability [40,41,42]. Such contradictory results led to the recognition that MFs may exert a whole spectrum of biological effects (from none through inhibitory/negative to stimulatory/positive), depending on the bacterial species analyzed, the nature of the emitted magnetic signals and the time of magnetic exposure [32,35,43]. Therefore, one of the explanations for the aforementioned discrepancies in the results obtained by different authors relates to the variety of the applied MF-generating systems, resulting in the diversity of the generated MFs, as well as to the multitude of species of microorganisms (or strains within a particular species) exposed to MFs [44]. Unfortunately, the above lack of methodological consistency in the research on the impact of MFs on microorganisms prevents this innovative approach from crossing the border separating in vitro analyses from preclinical studies. Therefore, in the present work, we attempted to take a step towards systematization of the knowledge in this area, focusing on only one type of MF, i.e., rotating magnetic field (RMF, a field where opposite poles rotate around a central point or axis), as well as on one species of bacteria (*S. aureus*) and one group of antibiotics (β-lactams).

Our research team has long-standing experience in studying RMF applications. We previously demonstrated the impact of RMF on the growth, metabolic activity and biofilm formation of several different species of microorganisms [20,22]. Moreover, in a distinct line of investigation, we analyzed the combined effect of RMF and different (non-β-lactam) antibiotics and antiseptics against *S. aureus* and *Pseudomonas aeruginosa* biofilms [10]. The obtained results indicated that the reduction of biofilms exposed to RMF and antimicrobials was 50% higher compared to biofilms exposed only to antimicrobials. We assumed that the observed effect could be related to the mixing effect caused by the RMF, which allowed antimicrobials to effectively reach deeper layers of biofilm.

The purpose of this study was to investigate the influence of the RMF on the changes in MRSA strains’ susceptibility to β-lactam antibiotics. We hypothesized that RMF could have an impact on the antibiotics’ activity and penetrability through the bacterial cell membrane, resulting in a higher rate of eradication displayed by the antibiotics. This assumption was backed up by the results of the experiments performed by Golberg et al. (2014) [45] and Tagourti et al. (2010) [46], where other types of electromagnetic fields (EMFs) were used to permanently damage cell membranes (presumably by membrane irreversible electroporation). Assuming that β-lactams can disturb cell wall synthesis in MRSA (even if these alterations are of minor character and do not lead to cell destruction), it can be expected that, together with RMF-induced alterations, a kind of booster effect may be observed. To investigate the above hypotheses we aimed to answer the following questions: is it possible to increase the susceptibility of MRSA strains towards β-lactam antibiotics in the presence of the RMF?; is the observed effect connected to the influence of the RMF on the antibiotic molecules, bacterial cells or both?; does the observed effect depend on the bacterial strain and apply to the entire group of β-lactams?; is the observed effect of a permanent character or it occurs only in the presence of the RMF? Furthermore, the associations between RMF parameters, as well as exposure conditions and the changes in antibiotic susceptibility, were analyzed.

## 2. Results and Discussion

### 2.1. Analysis of Changes in Antibiotic Susceptibility of S. aureus Strains under the Influence of RMF

The performed analyses confirmed that the bacterial strains used in the experiment belonged to *S. aureus* species. Resistance to cefoxitin (determining resistance to methicillin), and the presence of the *mecA* gene were confirmed for all analyzed MRSA strains. Accordingly, all strains which were included to the study as MSSA were susceptible to cefoxitin and did not harbor the *mecA* gene (Supplementary Figure S1).

The reference MRSA strain (ATCC 33591) and two clinical isolates (namely MRSA 1 and MRSA 2) were selected for the first phase of the analyses, which aimed to assess the changes in the aforementioned strains’ susceptibility to cefoxitin in the presence of RMF. Because resistance to cefoxitin indicates resistance to most other β-lactams, three other antibiotics from this group were included in this experimental line. The research previously conducted by our research group, as well as the studies of several other authors, revealed that the strength of MF impact (regardless of its type or the phenomenon analyzed in its presence) depends on exposure duration as well as on the intensity and/or the frequency of MF [19,20,32,43,47], because these two factors determine the physical characteristics of the magnetic signal [48,49].

It should be explained here that in the case of the RMF set-up used in the present study, the frequency of AC determines the MF intensity and, importantly, it is responsible for the physical characteristics of the magnetic wave shape. For this reason, the first analyses were carried out to identify the AC frequency at which the generated RMF induced the greatest changes in antibiotic susceptibility. As the optimal exposure time to the RMF has not been established at this stage of the experiment, seeded bacterial cultures on agar plates with the antibiotic were subjected to RMF for 18 h, because this is the time recommended by EUCAST (2021) [50] guidelines for antibiogram preparation. 

**Figure 1:**
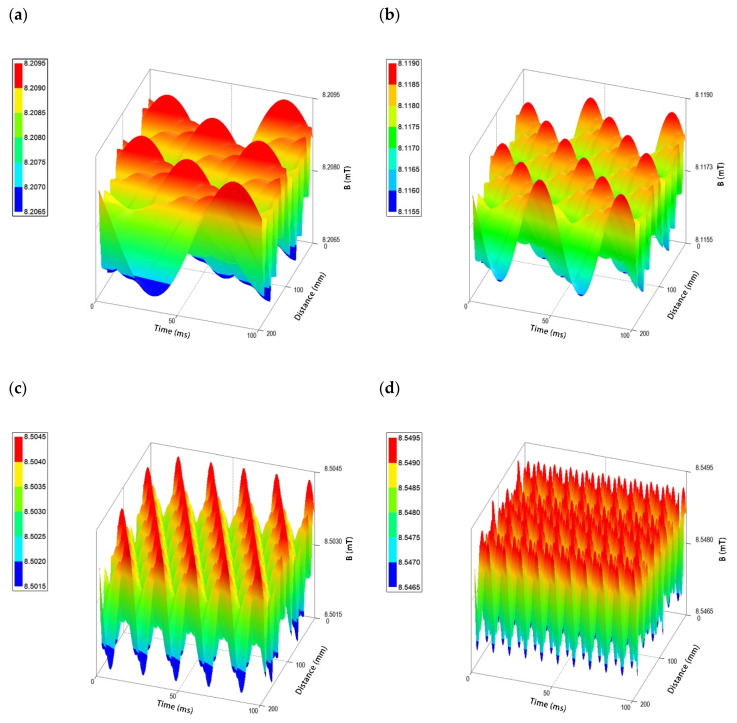
Changes of magnetic flux characteristic depending on the applied AC frequency: (**a**) 5 Hz; (**b**) 10 Hz; (**c**) 25 Hz; (**d**) 50 Hz.

It was shown that the strongest effect in terms of changes in growth inhibition zone diameter was observed at the lowest current frequency enabling the provision of the magnetic characteristic for the RMF (5 Hz) in the applied setup (Table 1). As shown by the simulative calculations, at 5 Hz the amplitude of the RMF was characterized by a longer period between magnetic induction maximal strength state (100 ms with *B*_max_ 8.1 mT). In contrast, a significantly lower effect in terms of the changes in antibiotic susceptibility was observed when RMF at 50 Hz (the highest current frequency in applied set up) was used. The RMF at this AC frequency was characterized by a shorter period, with 10 ms time between magnetic induction maximal strength state with *B*_max_ 8.5 mT (Figure 1). For the intermediate AC frequencies applied (10 Hz and 25 Hz), the changes in inhibition zones were also observed for most of the antibiotics included in the experiment, although the diameters of the zones were, in most cases, smaller, and in no case greater than those observed at the RMF generated at 5 Hz. 

When the MRSA 2 strain was exposed to RMF at 10 Hz in the presence of cefepime, cefuroxime and ceftriaxone, a zone of partial growth around the antibiotic discs was formed. This zone consisted of a bacterial layer, thinner and distinctly demarcated from the staphylococcal lawn grown on the rest of the agar plate (Supplementary Figure S2). It was confirmed that the discussed layer did not consist of contaminating species but of cells of the staphylococcal strain. A similar result (presence of partial growth zone around the antibiotic disc) was also observed when the same MRSA 2 strain was challenged against cefepime in the presence of RMF at 25 Hz. In turn, when RMF at 50 Hz and three of the aforementioned antibiotics were applied, the staphylococcal lawn formed by MRSA 2 reached the edge of antibiotic discs (no inhibition or reduced growth zones were noticed). It can be thus assumed that the observed effect could be related to RMF activity, because its frequencies (5, 25, 50 Hz) were the only variables in this experiment. However, no such effect of RMF was observed for MRSA 1 and ATCC 33591 strains. At 25 Hz RMF, the zones of their growth inhibition were clean and of similar size as the zones observed at 5 Hz. Such results may be related to the specific features of a given strain exposed to the RMF. It is well-known that sensing stress factors, bacteria switch on new pathways aimed at cell preservation [51]. In particular, the transposition, which represents an important source of genetic variability, can be induced in bacteria exposed to environmental stress, e.g. to the MF [49,52,53]. By this means, bacteria try to adapt using intra-strain variability. This allows enhancement of the persistence of bacteria and promotion of the selection of clones adapted to the particular stress conditions [54], e.g., RMF of specific characteristics. The conclusion which can be drawn from the discussed results is that RMF potential to induce changes in the antibiotic susceptibility of MRSA strains is not restricted only to the RMF generated at 5 Hz. Nevertheless, because the most favorable results were obtained at 5 Hz, further studies and analyses were performed in RMF using this frequency.

It is worth noting that in our previous work, a completely different pattern of changes in antimicrobial activity was observed with regard to the applied RMF frequency [10]. In the above work, we aimed to assess the activity of various antimicrobials (antibiotics and antiseptics) against staphylococcal and pseudomonal biofilms exposed to the RMF generated in the range from 10 to 50 Hz, in which the higher the frequency, the greater the antimicrobial effect observed. We assumed that this effect could be caused by the direct correlation between magnetic induction and mixing effect within the biofilm matrix, which was immersed in a liquid microbiological medium. Our assumption was that the more active the MF (please refer to Figure 1), the more particles of the antimicrobial could reach and deactivate bacterial cells within biofilm layers. These results are in opposition to those presented in Table 1. However, the previous study used not only a different biological model (biofilm) but also analyzed other antimicrobials. The above additionally emphasizes the impact of the applied variables on the final outcome of the experiment. The findings of the present study agree, in principle, with the report of Stepanian et al. (2000) [55] who revealed that the percentage of cell survival was proportional to the increase in EMF frequency. The highest percentage (53%) was observed at 50 Hz and the lowest (20%) at 4 Hz. In other words, the lower the frequency of the EMF, the lower the bacterial survival rate. Therefore, although the results of research of the effects of MFs on microorganisms frequently suggest that the effect is proportional to the intensity or frequency of the generated field, it should not be taken as a binding principle. Contradicting results may be explained, e.g., by the differences in MF wave characteristics at lower and higher frequencies, which act on the bacterial cells as a stress factor and consequently cause disturbances in their development; the exposed cells are alternately (however, with different frequency) subjected to weaker and stronger MF influences. As was already indicated, the amplitude of the RMF at a lower frequency is characterized by a longer period between magnetic induction maximal strength state, whereas at higher frequencies this period is shorter. Simultaneously, the applied AC frequencies (5 Hz https://youtu.be/YXH5CkArdQ0, accessed on 1 September 2021, 10 Hz https://youtu.be/4TGUOQOVOLo, accessed on 1 September 2021, 25 Hz https://youtu.be/8r1AcS0dIP0, accessed on 1 September 2021, 50 Hz https://youtu.be/gfr7AYqCyh8, accessed on 1 September 2021—please click the links to watch the simulation) generated magnetic flux rotation around the stator with different synchronous speeds of 150 rpm, 300 rpm, 750 rpm and 1500 rpm, respectively (calculations performed on the basis on the manufacturer’s characteristics of the stators).

Previous studies have also indicated the ability of different MF types to disturb microbial structures and to permanently damage cell membranes, presumably by their irreversible electroporation [45,46]. Fojt et al. (2004) [32] explained that a drop in bacterial viability after exposure to MF was caused by an increase in the permeability of ion channels in the cytoplasmic membranes or by the formation of free radicals in bacterial cells. The relationship between the changes in the induction of the MF and the formation of active oxygen and free radicals in bacterial cells was previously demonstrated by Kohno et al. (2000), Fojt et al. (2004) and Jin et al. (2009) [32,56,57]. Therefore, it can be expected that in the case of a fairly homogeneous MF, e.g., the RMF generated at 50 Hz, which is characterized by relatively low MF strength fluctuations, bacteria could adapt more easily to the stressor. Such a mechanism was previously reported by Mittenzwey et al. (1996) [58] who showed high resistance of different bacteria to MFs due to the intracellular repair systems and self-regulatory mechanisms. The data reported by the above-mentioned researchers, together with the results presented in Table 1 and Figure 1, indicate the possibility of an additive interaction between β-lactam antibiotics and RMF. At this stage of the experiment, we assumed that the potential mechanisms behind the observed interaction may include a direct influence of the RMF on antibiotic molecules, bacterial membrane/cell wall or metabolic intercellular processes.

The next stage of the experiment aimed to determine the optimal RMF exposure time to obtain the highest increase in antibiotic susceptibility. Previous papers of our research group [19,20] as well as reports of other authors [44,59], indicated that the time of magnetic exposure (apart from the intensity, frequency and characteristics of the field wave) is also of key importance with regard to the effect exerted on biological systems. Depending on the exposure time, the MF may have a different effect on bacterial viability, i.e., it may increase it [40,41,42] or reduce it [32,34,35]. In the current study, the cultures with antibiotic discs were exposed to the RMF for a specified time, ranging from 1 to 12 h, and then the plates were transferred to the incubator until the 18 h period of incubation was completed. It was revealed that the zones of bacterial growth inhibition increased with the length of exposure time up to 12 h in most cases (Table 2). However, in the case of MRSA 1 exposed to ceftriaxone and MRSA 2 exposed to cefuroxime, the increase of growth inhibition stopped after 5 h of magnetic exposure. In turn, the first increase in the diameter of the growth inhibition zones around the antibiotic discs was observed after 2 h of the strain’s exposure to RMF compared to an unexposed strain. This effect was observed in *S. aureus* strains ATCC 33591 and MRSA 2 subjected to the activity of cefoxitin, cefepime and ceftriaxone. Nevertheless, in most cases the differences in inhibition zones were visible after 4 h of exposure to the RMF. It should also be explained here that in each of the above cases, the period of exposure to the RMF was shorter than the time needed to observe bacterial growth on the medium. However, in the case of cultures exposed longer, at least by the time after which bacterial growth on the media was already observed (>6 h), it was possible to measure the zones of growth inhibition even before transferring the plates to the incubator. It was found that in cultures exposed for more than 6 h, after further incubation w/o RMF, the inhibition zones were only slightly reduced (<2 mm) compared to the zones measured immediately after exposure to the RMF (Supplementary Table S1). It was also noticed that in a few cases, after magnetic exposure lasting for less than 6 h, a small number of individual colonies was also observed within the growth inhibition zones (thus they were referred to as zones of partial growth inhibition) (Supplementary Figure S3). No differences between the zones of growth inhibition measured immediately after RMF exposure compared to the zones remeasured after further incubation w/o RMF was observed only when the exposure lasted at least 10–11 h (depending on the staphylococcal strain), when the cultures were already well-developed (Table 2). For this reason, the optimal RMF exposure time ensuring the possibility of observing a stable effect was defined for 12 h.

**Figure 2:**
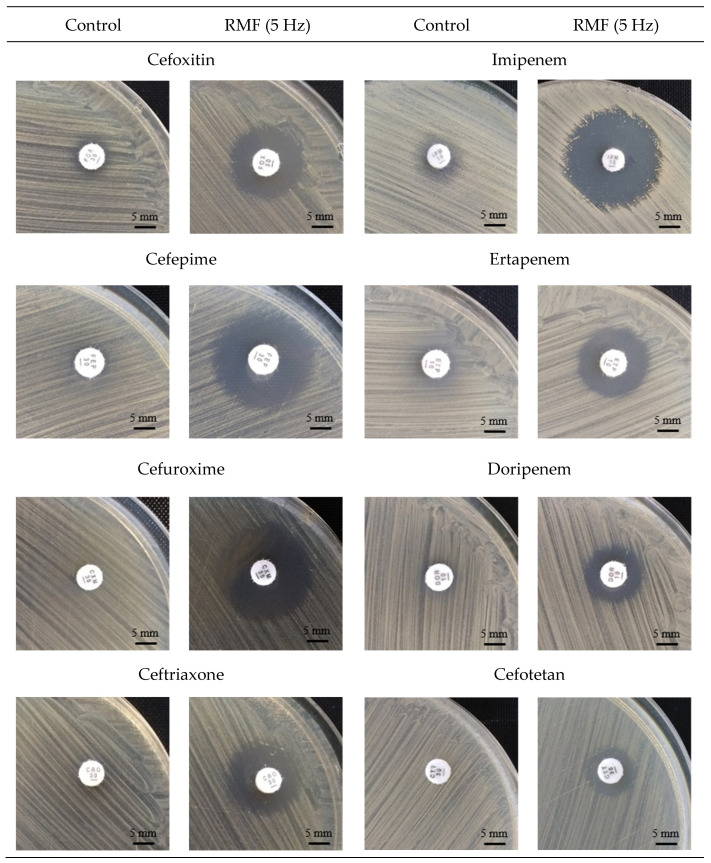
Representative pictures of growth inhibition zones (mm) in control and RMF-exposed (5 Hz) MRSA 1 cultures around discs with β-lactam antibiotics.

The next part of the experiment focused on changes in the susceptibility of the RMF-exposed bacteria to the various β-lactam antibiotics, including different classes of cephalosporins, carbapenems and penicillins. Although the general mechanism of action of β-lactam antibiotics is similar, there are some differences in their specific activity, e.g., related to different binding sites with the PBP2a protein or the binding energy value [60]. Harrison et al. (2019) [61] demonstrated that aminopenicillins (e.g. amoxicillin) bind better to PBP2a than cephalosporins. The size of the particles of individual antibiotics, as well as their charge, may also be important, especially taking into account the possible effects of the MF on charged particles, and thus on the process of their diffusion in the microbiological medium [62,63]. Our findings showed that an increase in antibiotic susceptibility in all RMF-exposed cultures was obtained for seven out of the eleven β-lactam antibiotics included in the experiment (Table 3, Figure 2, Supplementary Figure S4). A lack of increase in the diameters of growth inhibition zones in RMF was found only for ceftazidime and amoxicillin (for all three strains analyzed); cefradin (in the case of ATCC 33591 and MRSA 2 strains) and cephalexin (MRSA 2 strain). Unfortunately, we were unable to establish any pattern explaining the relationship between the properties, mechanism of action of the specific types of antibiotics and the observed changes in the zones of growth inhibition under the influence of RMF.

**Figure 3:**
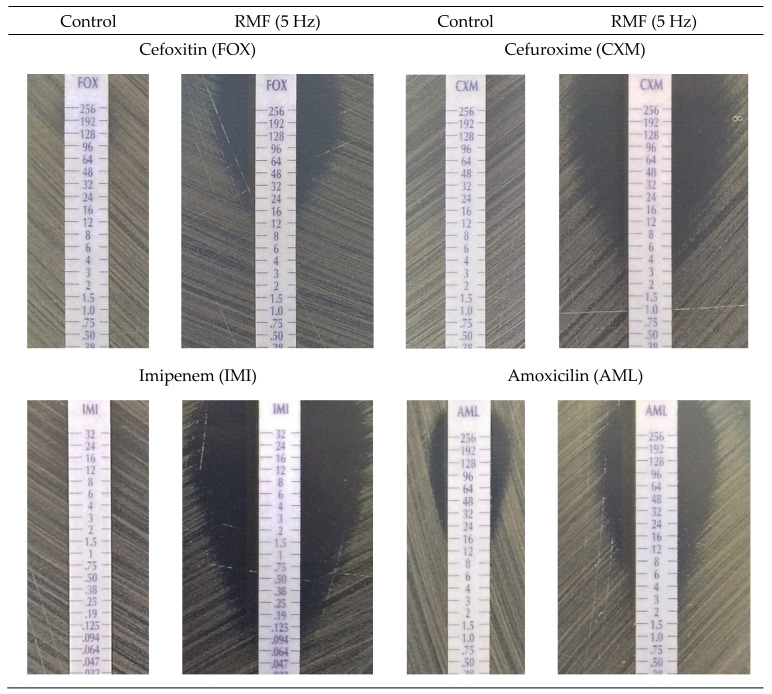
Representative pictures of gradient MIC strips (E-tests) with β-lactam antibiotics in control and RMF-exposed (5 Hz) cultures of the MRSA 1 strain.

The next part of the study aimed to determine whether the observed effect of increased sensitivity of β-lactam antibiotics caused by RMF exposure depends on the specific concentration of antimicrobial used. Since the disc diffusion method is limited to only one concentration of the antibiotic/disc, for the purposes of this experiment, gradient MIC strips (E-tests) were used. The use of E-tests enabled assessment of the changes in antibiotics susceptibility of bacterial cultures exposed to the RMF (5 Hz) depending on the concentration of the antimicrobials. Eight antibiotics were selected for the study, for which in the disc diffusion test the greatest and the lowest differences in the zones of growth inhibition compared to the unexposed control cultures were obtained. It was found that in each (except one) of the RMF-exposed cultures, a substantial decrease in the MIC value (by at least one order of the antibiotic concentration value marked on the E-test strips) occurred compared to the controls (Table 4, Figure 3, Supplementary Figure S5). The only exception was the RMF-exposed *S. aureus* ATCC 33591 cultures with ceftazidime, for which the MIC value was the same as for the control. In turn, the decrease in MIC values of cefoxitin, ceftriaxone, cefuroxime, ceftazidime and cefepime was particularly significant in the case of MRSA 1 and MRSA 2 strains exposed to RMF. The MIC values measured in the control settings were 256 µg/mL, while in the RMF-exposed cultures, MIC values ranged from 6 to 96 µg/mL, depending on the strain and antibiotic. Importantly, the use of more precise E-test methodology allowed to detect the effect displayed by RMF in the case of these antibiotics for which no or minimal changes in growth inhibition zones were observed when the disc diffusion method was applied. These antibiotics were, e.g., amoxicillin (in all RMF exposed cultures) and ceftazidime (in MRSA 1 and MRSA 2 cultures but not in the ATCC 33591 strain). To summarize this part of the results, it should be noted that the lack of the influence of the RMF, observed in the case of several antibiotics in the disk diffusion assay, was related to a low concentration of these antimicrobials in the test discs. Nevertheless, the exposure to RMF did not change the susceptibility level of the ATCC 33591 strain to ceftazidime. This reveals not only the importance of intra-species variability in their answer to the same stimuli but also the necessity of testing a high number of microbial strains to draw proper conclusions from the observed phenomena. 

**Figure 4:**
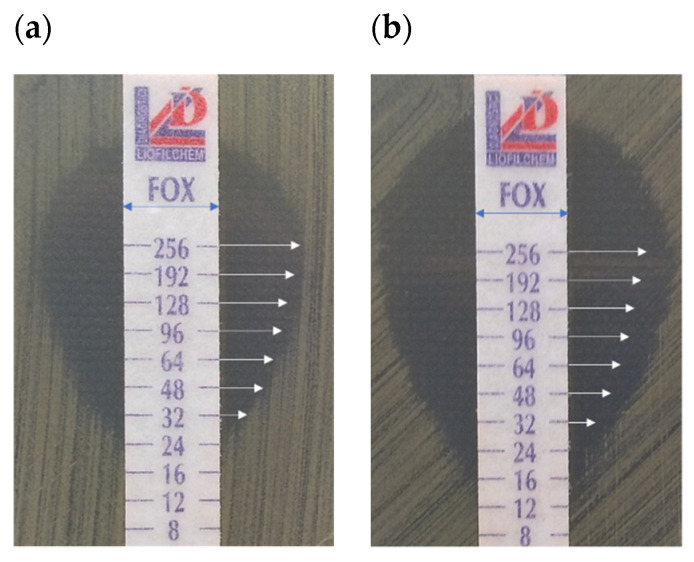
Representative pictures of growth inhibition zones around E-test strips with cefoxitin in (**a**) control and (**b**) RMF-exposed (5 Hz) *S. aureus* ATCC 33591 cultures. The arrows at a certain antibiotic concentration represent sections of the same length.

The additional finding from the antibiotic diffusion-based assays was the observation that the zones of growth inhibition obtained in the RMF-exposed cultures retained their characteristic shape of a circle in the disc diffusion method and an ellipse when the MIC strips were used (Figure 2, Figure 3, respectively). Importantly, in the case of E-tests, the zones of growth inhibition measured in a straight line from the edge of the strip across to the edge of the bacterial lawn in the RMF-exposed cultures were larger than in the control settings, approximately in the same manner as was previously observed when the disc diffusion test was applied (Figure 4). This observation is particularly important in the context of the basic methodological assumptions for the applied diffusion-based tests. A demonstration of the disturbances in the diffusion of antibiotics under the influence of RMF at this level of analysis would significantly hinder the correct interpretation of the obtained results. On the other hand, if such a phenomenon occurred, it would provide precious data on a possible interaction of RMF with antibiotics.

To determine whether the observed effect of elevated antibiotic susceptibility in RMF is strain-specific, the research was extended to include another 21 MRSA strains. It is well-established that the MRSA mechanism is mediated by the expression of an alternative of PBP2 protein (called PBP2a) characterized by a low affinity for β-lactam antibiotics, resulting in resistance to most β-lactams. However, PBP2a encoded by the *mecA* gene which is carried on a mobile genetic element known as a staphylococcal cassette chromosome *mec* (SCC*mec*) can be regulated by two independent regulatory systems (*mecI*-*mecR*-*mecR2* and *blaI*-*blaR*) and multiple chromosomal genes. As shown by other authors, also PBP1-4 and PBP2a structures are not identical between staphylococcal strains [60]. Moreover, β-lactam resistance in most MRSA is heterogeneous, meaning that while most cells in a population have low MICs, some fraction can survive at much higher MICs [64]. Despite the naturally occurring differences between strains and their different susceptibility to cefoxitin (confirmed on the basis of the results obtained in control cultures), our studies showed that all analyzed MRSA strains displayed elevated susceptibility to cefoxitin (Table 5). In the case of eight of the 20 strains, the diameter of the inhibition zones increased at least by 10 mm, while in the others by at least 4 mm. Of note was the observation that the smaller the zone of growth inhibition under the control conditions, the greater the change in its diameter as a result of RMF exposure.

The next stage of the study aimed to demonstrate whether the changes in susceptibility to β-lactam antibiotics are related to the MRSA mechanism based on the presence of PBP2a protein (whether the effect of changes in susceptibility to β-lactams is observed only in MRSA strains, while in the case of MSSA strains, the changes are not observed) or whether the observed effect is strictly related to the presence of the antibiotic in the microbial culture (changes in antibiotic susceptibility occur in the presence of the antibiotic in the RMF-exposed culture, regardless of the lack of methicillin resistance mechanism). For the purposes of this research, five *S. aureus* strains lacking the *mecA* gene and showing susceptibility to methicillin in a phenotypic test with the cefoxitin-saturated disc were used (Supplementary Figure S1). The findings did not show any changes in the diameters of growth inhibition of RMF-exposed (5 Hz) cultures when the disk diffusion test was applied (Table S2). However, when E-tests were used, for all analyzed MSSA strains differences in MIC values were found between the control and RMF-exposed cultures with cefuroxime and cefepime and, in the case of four strains cultivated with ceftriaxone (in all cases, by one order of concentration value) (Table 6). In turn, no differences were found for cefoxitin. Therefore, it can be assumed that the observed changes in susceptibility to β-lactam antibiotics observed in the case of MRSA strains under the influence of RMF may, at least to some extent, be related to the presence of the alternative PBP2a protein. On the other hand, it can also be noted that the MIC values in the control cultures were relatively low; therefore, it cannot be excluded that the effect of RMF concerning the changes in antibiotic susceptibility has its limitations in the case of strains showing high susceptibility to the analyzed antibiotics.

### 2.2. Effect of RMF on Changes in Number of Culturable Bacteria

**Figure 5:**
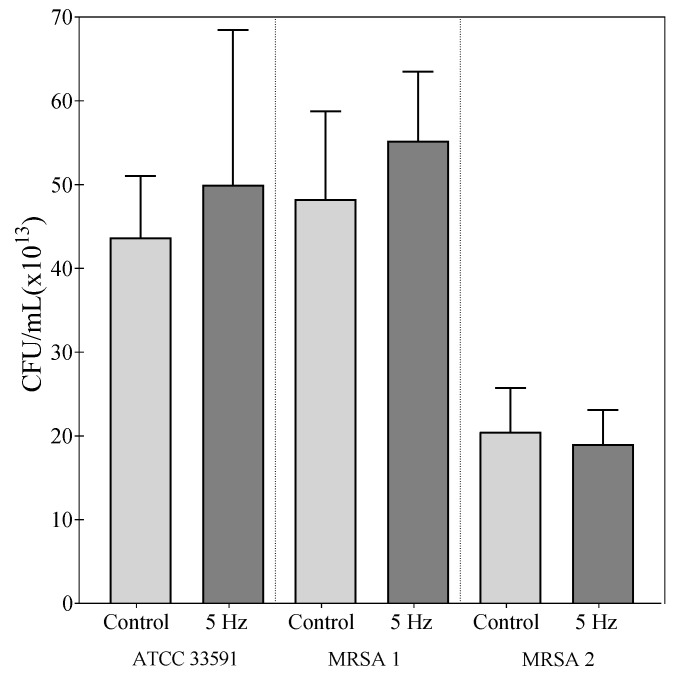
The number of culturable bacteria, expressed as a CFU/mL, after 12 h of RMF exposure (5 Hz) and in control, unexposed setting. The results are presented as a mean ± SEM calculated using six values (three from each biological replicate). The results show no statistically significant differences at *p* < 0.05.

The conducted studies also included an assessment of the influence of the RMF on the changes in the number of bacterial cells having ability to form colonies on the M-H agar. The study was performed to detect the possible bactericidal impact of RMF exposure. Although there are numerous literature data presenting the influence of MFs on the growth and viability of bacterial cells [21,32,56,65], the obtained results did not show any significant differences in the CFU number in RMF-exposed vs. control settings (Figure 5). There were also no differences found in the morphology or size of bacterial colonies formed (Supplementary Figure S6). However, in the context of the current work, it is worth noting that our previous research [19,20,22] and the research of most other authors (e.g. Kohno et al., 2000 [56]; Kermanshahi and Sailani, 2005 [65]) were carried out with the use of bacterial cultures in liquid media. Therefore, considering our previously published data regarding, e.g., mixing efficiency under the influence of the RMF [63], it can be noticed that the physical phenomena occurring under the influence of the MFs significantly differs depending on the type of the medium in/on which the bacteria are cultivated during exposure. It should therefore be concluded that for the purposes of the present study, the most important finding was the absence of a bactericidal effect of the RMF on bacterial cells.

### 2.3. Effect of RMF on Changes in Relative Number of Live and Dead Bacterial Cells 

The data presented in Figure 5 show that the number of culturable bacteria did not differ between RMF-exposed vs unexposed cultures. Nevertheless, the results of quantitative culturing did not provide data on the physiological state of the cells after exposure. Therefore, in the next assay, RMF exposed and unexposed bacterial cultures were stained with a combination of propidium iodide and SYTO9 dyes and visualized by means of fluorescence microscopy. This approach allows not only to distinguish live (dyed green) from dead (dyed red) cells, but also to show changes in the structure of the bacterial cell wall because of the properties of the dye components, which are able to penetrate these cells of bacteria which are living but display a compromised integrity of the cell wall [66]. The further post hoc processing of fluorescent pictures allowed us to change the intensity of red/green fluorescence into a value referred to as Mean Grey Value (MGV) and, using this parameter, to analyze the changes in the relative number of live (noncompromised) and dead (compromised) cells exposed or unexposed to the RMF. 

**Figure 6:**
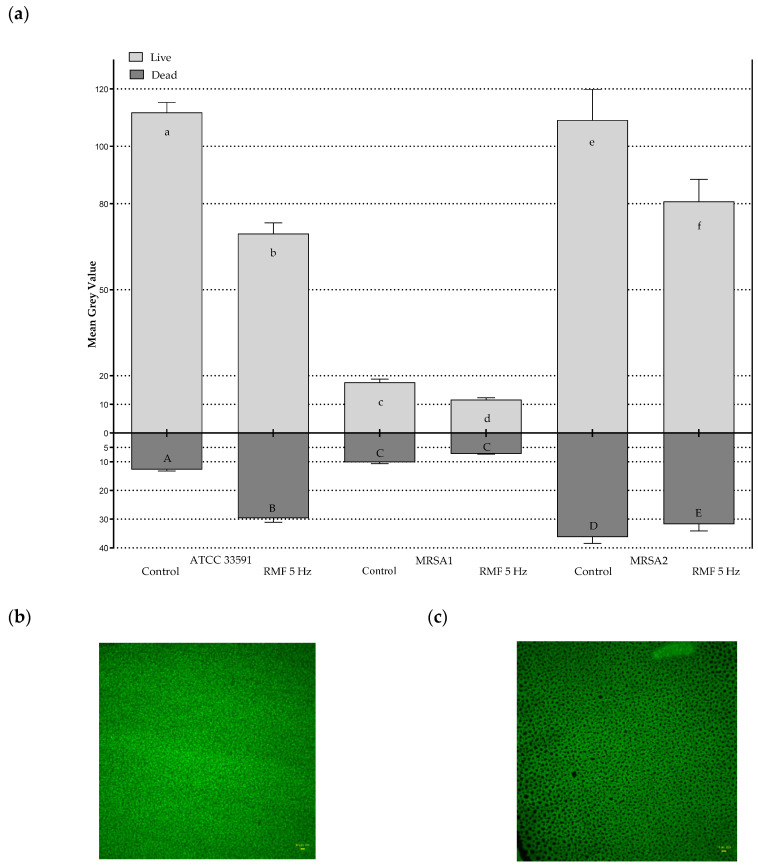
(**a**) The average intensity of fluorescence of dead and live MRSA cells after 12 h of RMF exposure (5 Hz) and in the control, unexposed setting, presented as differences in Mean Grey Value (MGV). (**b**) Picture of dyed with SYTO9 staphylococcal ATCC 33591 cells unexposed and (**c**) exposed to RMF (5 Hz). The results presented in (**a**) are shown as a mean MGV ± SEM calculated using 24 Regions of Interest (ROIs) obtained from four biological replicates. Different letters indicate statistical differences (*p* < 0.05) between RMF-exposed and unexposed cultures of the same staphylococcal strain.

The results presented in Figure 6a show significantly higher intensity of green fluorescence (expressed as MGV), corresponding to the number of live (noncompromised) cells in the cultures unexposed (Figure 6b) compared to the cells exposed to RMF at 5 Hz (Figure 6c). This phenomenon was observed for all investigated MRSA strains. Noteworthy is that the MGV measured for live/non-compromised cells in the control setting differed between strains (with an average MGV value of 111.83 vs. 17.83 vs. 109.14 for strain ATCC 33591, MRSA 1 and MRSA 2, respectively), showing species-specific differences in the ability to multiply on an agar surface. Noteworthy is that the average reduction in the relative number of live cells (calculated from the mean value from the 3 strains analyzed) being the result of exposure to the RMF was 32.64 ± 6.07% (the MGV value recorded for unexposed strains was considered 100%). The relatively low standard deviation of the mean reduction value shows that, regardless of the strain applied and its ability to multiply on agar, a comparable relative number of cells was affected by the activity of the RMF to the level which can be measured by the applied technique. During the 12 h of exposure to the RMF, the seeded cells form multilayered aggregates on the agar surface and display differentiated properties regarding their metabolic activity and division rate. As was already explained, in this manner, bacteria can try to adapt through intra-strain variability, so that better adapted variants can persist [54]. Such an explanation is in line with the observation of single colonies or partial growth inhibition zones when the antibiotics disc diffusion test was applied. It can also be hypothesized that the reduction in the intensity of green fluorescence (expressed as a drop of MGV) caused by exposure to the RMF, should correlate with a synchronic boost in the number of dead (dyed with propidium iodide) cells. Nevertheless, such a phenomenon occurred only in the case of the ATCC 33591 strain, which displayed the highest value of MGV in the control setting among the analyzed microorganisms (Figure 6a). In the case of MRSA 1 and MRSA 2, the opposite trend was observed (although devoid of statistical significance), namely a drop in red signal intensity coming from the dead (compromised) cells in RMF-exposed cultures. Such a fact may be caused by specific methodological stages applied for L/D dyeing. Propidium iodide (red dye) is incorporated into cells with compromised membranes (dead cells or damaged cells). However, the subsequent stages of rinsing and centrifugation performed during the dyeing procedure cause further damage to the already altered cells and, in effect, lead to their removal (together with the incorporated propidium iodide dye) from the reaction environment. By contrast, the green fluorescence of the SYTO9 dye is microscopically recorded only in live cells (of intact cell walls). Because such cells are less prone to damage, due to rinsing/centrifugation procedures, this results in their higher share (comparing to the cells dyed with propidium iodide) in the pellet later applied for microscopic analyses [67]. 

### 2.4. Effect of RMF on Diffusion of Antibiotics

This line of investigation was performed to analyze another variable in the applied experimental system, namely the differences in antibiotic diffusion which may potentially occur between bacterial cultures exposed and unexposed to the RMF. Numerous literature data [32,62], as well as the previous experience of our research group [63] indicated that RMF influence was related to its interactions with electrically charged molecules. Therefore, we aimed to investigate whether magnetic exposure alters the diffusion of the antibiotic in the agar medium. The findings obtained in this part of the study were especially important for the proper interpretation of the results obtained by means of the disk-diffusion method and E-tests. Noteworthy is that the results obtained from the application of the above-mentioned tests allowed drawing a preliminary conclusion that, during exposure to the RMF, the diffusion of antibiotics in the agar medium was not significantly altered. Specifically, the zones of growth inhibition (in the disk-diffusion method) maintained their oval shape, while the zones in the E-test method remained symmetrical on both sides of the strip soaked with the antibiotic gradient (Figure 2, Figure 3 and Figure 4). 

**Figure 7:**
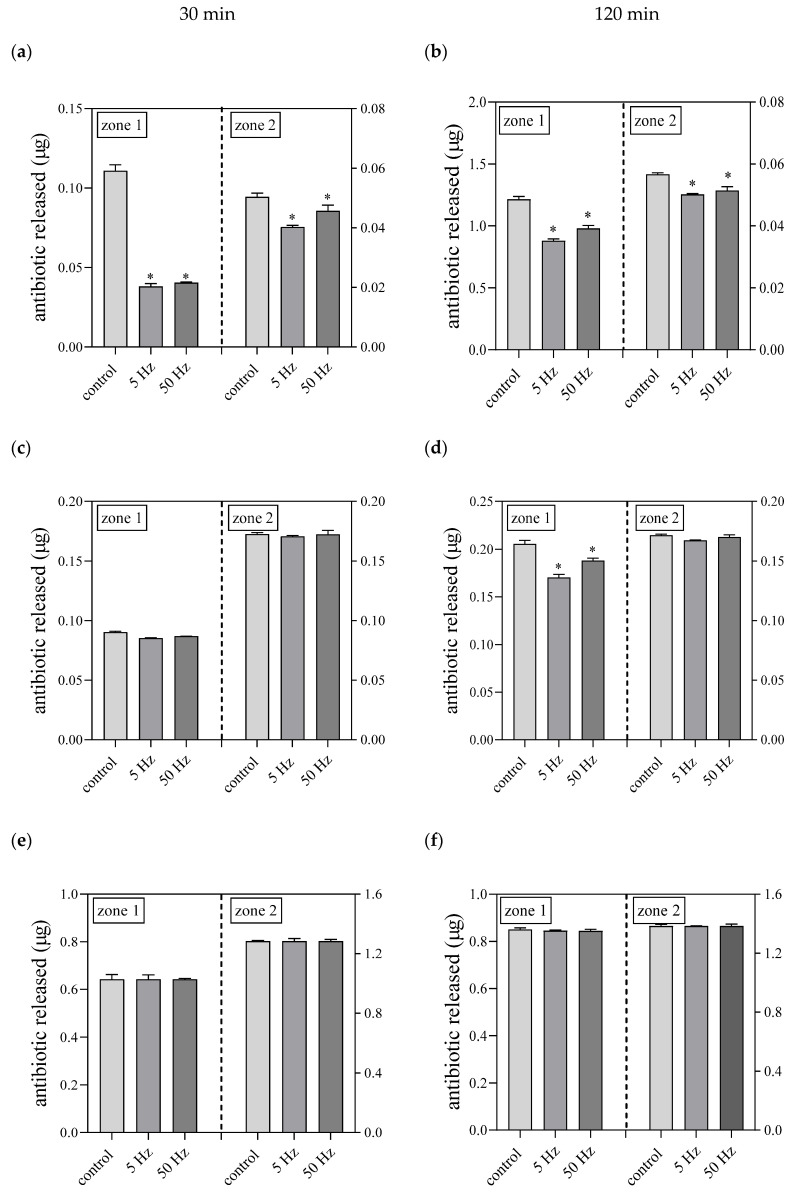
Concentrations of antibiotics in agar in control and RMF-exposed (5/50 Hz) settings; (**a**,**b**) cefoxitin; (**c**,**d**) cefepime; (**e**,**f**) imipenem. The results are presented as mean ± SEM calculated using six values (three from each biological replicate). *—indicates statistical differences (*p* < 0.05) between control and RMF-exposed settings.

For a more detailed analysis of the potential influence of the RMF on antibiotic diffusion in the agar medium, the concentrations of these antimicrobials in specific parts of the agar plate were measured using the LC-MS/MS method (Supplementary Figure S7). The obtained results did not show any increase in the concentration of antibiotics in the agar due to the RMF exposure (Figure 7). On the contrary, even lower concentrations of antibiotics were detected in the samples cut out from the RMF-exposed agar as compared to the unexposed control. As mentioned before, in our previous work we demonstrated the influence of the RMF on mixing efficiency [63,68]. However, such tests were always carried out in liquids (including liquid microbiological media), while in the case of the analyses performed in the current study, the diffusion took place in 1.7% agar. Thus, it can be assumed that the application of a relatively weak MF (*B*_max_ 8.3 mT at 5 Hz and *B*_max_ 8.5 mT at 50 Hz) did not act on the antibiotic particles strongly enough to relocate them significantly through the pores of the agar gel.

### 2.5. Effect of RMF on Cell Morphology

**Figure 8:**
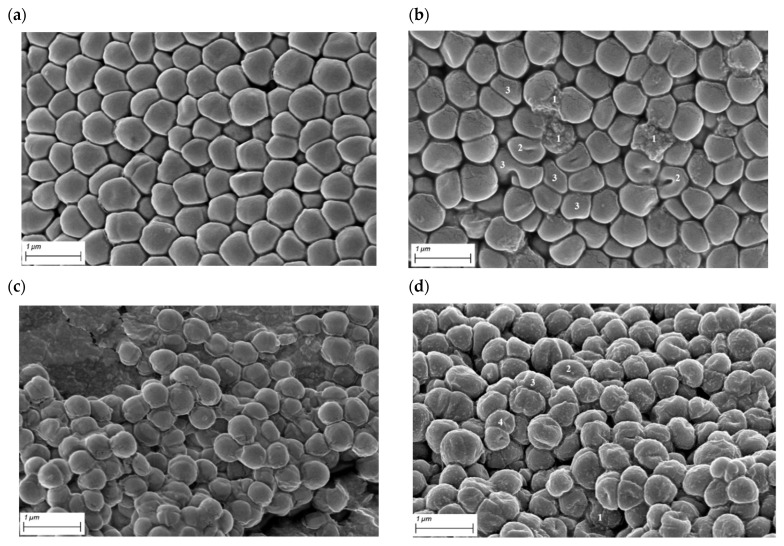
Scanning electron microscopy images: (**a**) MRSA 1 cells unexposed to the RMF; (**b**) MRSA 1 cells exposed to the RMF (5 Hz); (**c**) MRSA 2 cells seeded on an agar plate in the presence of a disc containing cefoxitin; (**d**) MRSA 2 cells seeded on an agar plate in the presence of a disc containing cefoxitin and exposed to the RMF (5 Hz). 1—cellular content leakage; 2—collapse of cell wall; 3—strong deformation of cellular shape, 4—cell size reduction. Scale bars represent 1 µm.

In the present study we confirmed that the observed RMF effect is related to changes in antibiotic susceptibility but not to the direct bactericidal feature of the RMF (measured by means of quantitative culturing). Moreover, we showed that the presence of RMF does not correlate with changes in the diffusion of antibiotics in the agar medium. The above results encouraged us to perform analyses capable of detection of such subtle phenomena caused by RMF as minor cell damage that could weaken the cell structure without completely destroying it. RMF-exposed cells displayed significant alterations compared to their unexposed counterparts. These changes included mostly the shape and size of cells; also collapse of cell walls and cellular leakage was observed (Figure 8a,b, Supplementary Figure S8). Noteworthy is that with the exception of the last type of alteration, of a rather irreversible character, one may assume that changes in such altered cells as those pictured in Figure 8a (and marked with numbers 2 and 3) could be reversed [69] if the cells were seeded in a fresh microbiological medium and cultivated without RMF presence. Such a phenomenon largely explains the lack of changes in the number between cultivable bacteria exposed and unexposed to RMF (Figure 5) and a drop in live cells (dyed with SYTO9) in the RMF-exposed vs unexposed setting (Figure 6). 

The results related to durability of the effect of exposure to RMF (post-exposure effect) showed that bacterial cultures previously exposed to RMF behaved in the same way as the control unexposed cultures, i.e., the antibiotic susceptibility level was the same for both types of cultures (Supplementary Table S3). Thus, despite the inevitable biases related to the applied methodology, the results of this part of the investigation suggest that RMF acts by partial disintegration of staphylococcal cell walls. This statement is particularly important in the context of issues presented in this article (Table 1, Table 2, Table 3, Table 4, Table 5 and Table 6) because it satisfactorily explains the increased efficacy of β-lactam antibiotics acting against already weakened (as a result of RMF exposure) staphylococcal cell walls. Therefore, another SEM analysis was performed to visualize this hypothetical, boosting effect of the RMF on cefoxitin activity. Figure 8c shows staphylococcal population (resistant to cefoxitin) grown on agar with the aforementioned antibiotic applied. Figure 8d shows the same resistant staphylococcal strain grown on agar with cefoxitin introduced, but additionally exposed to the RMF (5 Hz). It can also be seen that the application of the RMF together with cefoxitin translated into explicit morphological changes of staphylococcal cells, including alteration of cell shape, collapse of cell wall, decreased turgor and reduced size (probably effected by cytoplasmic leakage). Noteworthy is that these changes were more ubiquitous in the discussed setting (RMF + antibiotic) than in a setting when only RMF was applied (please compare Figure 8a with Figure 8d).

**Figure 9:**
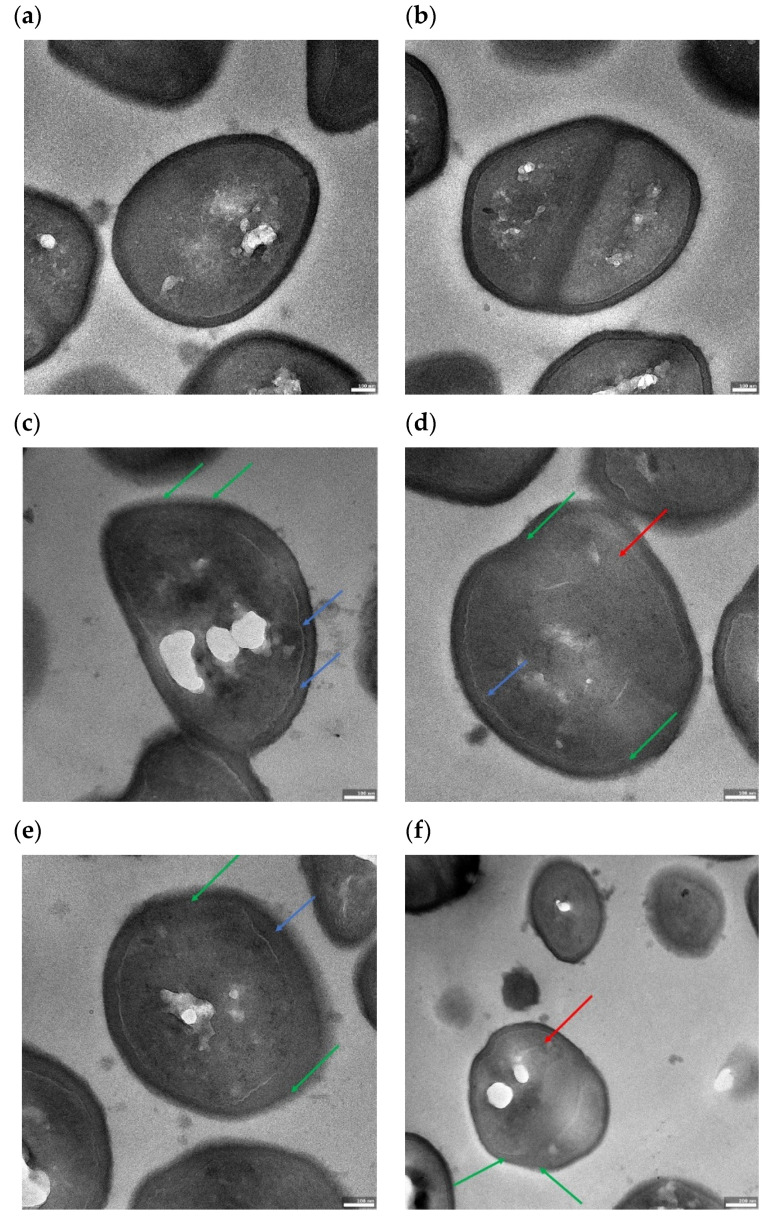
Transmission electron microscopy cross-section of (**a**,**b**) MRSA 1 cells unexposed to the RMF; (**c**–**f**) MRSA 1 cells exposed to the RMF (5 Hz). Red arrows indicate deformation of shape of staphylococcal cells after exposure to RMF. Green arrows indicate loss of cell wall density after exposure to RMF. Blue arrows indicate contraction of cytoplasm after exposure to RMF. Scale bars represent 100 nm (picture **a**–**e**) and 200 nm (picture **f**). Magnified regions of interest are additionally presented in Supplementary Figure S9. The cellular shapes of ca. 400 nm diameter seen in the image 9f are staphylococcal cells cut in their apex parts.

To get additional data concerning RMF impact on staphylococcal cell morphology, cross-sections of bacterial cells (exposed and unexposed to the RMF) were performed using transmission electron microscopy. In Figure 9 and Supplementary Figure S9, representative pictures of the clinical strain MRSA 1 are presented. Pictures a,b of Figure 9 show bacterial cells unexposed to RMF, with an oval shape typical for staphylococci, whereas the RMF-exposed staphylococcal cells present deformed morphology, particularly well-visible in pictures c,d of Figure 9. The deformation pattern included cell elongation (loss of oval shape, picture c of Figure 9) or formation of bulges (indicated with red arrows in Figure 9d,f). As seen in Figure 9a,b, the walls of unexposed cells are evenly contrasted and strongly distinguished from the cytoplasm, while in RMF-exposed cells a lower contrast was observed between the cytoplasm and the cell wall (marked with green arrows in Figure 9), indicating a loss of wall density. In the case of RMF-exposed bacteria, contraction of the cytoplasm is also visible, manifested by an uneven border between the cytoplasm and the cell wall (blue arrows in Figure 9) suggesting lower cytoplasm density. Such a cytoplasmic condensation induced by membrane damage was recently observed in Gram-negative *E. coli* by other authors [70]. The above-mentioned observations suggest that RMF activity translates into a reduction in cell wall density, which in turn manifests itself by cell shape deformations and (at least) partial leakage of cytoplasm.

The following hypothesis is thus worth considering. As already mentioned, MRSA strains carrying the *mecA* gene encoding an alternative form of PBP, called PBP2a, have a reduced (to a varying extent) affinity for β-lactam antibiotics [61,71]. Therefore, PBP2 and PBP2a are not the same in different strains (the polymorphism of the *mecA* complex may affect the function of these genes and methicillin resistance mechanism) and thus these proteins can show different affinity for the same β-lactam antibiotic. The EUCAST defines the presence of methicillin resistance when the inhibition zone is ≥22 mm, not only when there is no inhibition zone. This means that binding of β-lactam antibiotics to PBP2a protein occurs and translates to a certain level of inhibition of peptidoglycan synthesis in MRSA strains. The process of binding, and the aforementioned inhibition, are significantly less effective compared to MSSA strains expressing unmodified PBP protein, as well as compared to the competitive reaction with peptidoglycan chains. Thus, given the competitive mechanism, a large proportion of the PBP2a is not blocked and is involved in the cross-linking of the peptidoglycan chains, and so the cell survives (at least in relatively low concentrations of the β-lactam antibiotic). Considering the observations using SEM, TEM and fluorescence microscopy suggesting that the RMF induced disturbances in the structure of the bacterial cell wall, it can be assumed that the small amount of β-lactam antibiotic, which blocks the activity of some of the entire PBP2/PBP2a protein present in the MRSA cell, is sufficient to induce further structural changes in the cell wall, which, in effect, makes it impossible to maintain the intracellular osmotic pressure and leads to cell disintegration. Additionally, considering one of the RMF interaction mechanisms associated with increasing the mixing efficiency [63], which apply to both the external and internal environment of the cell culture and cells, it can also be assumed that in the RMF-exposed bacteria there is an additional increase in the pressure of the external and intracellular liquid on the cell wall that may lead to its further damage.

## 3. Conclusions

The presence of MF is one of the most basic properties of our physical reality. It escapes our sensual experience and can be described only by the use of mathematical formulas. Noteworthy is that the specific RMF created for the purposes of this study, has no reflection in the natural environment. It is a type of stimulus unknown (contrary to the geomagnetic field) to the bacterial pathogens we challenged by its means. The results of our study indicate that the analyzed reference and clinical *mecA*-positive MRSA isolates were prone to this stimulus, which manifested itself in the substantially elevated susceptibility to most of β-lactam antibiotics. By means of differentiated techniques and approaches we were able to indicate that this susceptibility is most likely related to RMF-induced changes in staphylococcal cell walls. These alterations were subtle enough to be missed when basic microbiological techniques (e.g., quantitative culturing) were applied. However, they were revealed by means of, among others, SEM or TEM microscopy. Nevertheless, the data obtained by means of basic microbiological techniques allowed refinement of the spectrum of possibilities standing behind the observed phenomenon and brought us to the conclusions presented above. 

The constantly growing number of methicillin-resistant strains (of nosocomial and community origin) warrants the search for new treatment options. In the light of the results presented in this study, the application of the RMF together with β-lactam antibiotics could be a promising direction to follow. Taking into account the high clinical need for the provision of new therapeutic options effective against methicillin-resistant strains, the data presented in this study have high developmental potential and could provide the basis for new treatment options for MRSA infections.

## 4. Materials and Methods

### 4.1. Microorganisms

Two reference staphylococcal strains (one MRSA-American Type Culture Collection (ATCC 33591) and one MSSA (ATCC 6538)) and two clinical MRSA isolates (MRSA 1 and MRSA 2) were used for experimental purposes. In the course of the investigation, an additional 24 clinical isolates including 19 MRSA (MRSA 3–MRSA 21) and 5 MSSA (MSSA 1–MSSA 5) were used in specific experiments. All analyzed clinical isolates belonged to the Strain Collection of the Department of Pharmaceutical Microbiology and Parasitology of Wroclaw Medical University. These strains originated from chronic wounds of patients treated in the Teaching Hospital of Wroclaw Medical University (Wroclaw, Poland). The strains’ species affiliation was confirmed in the first step by macroscopic observation of the specific colonies (yellow/golden oval shapes with distinct β-hemolysis zones) on Columbia agar (Graso Biotech, Jablowo, Poland). Then, the colonies were transferred into Müller-Hinton agar (M-H, Graso Biotech, Jablowo, Poland) and analyzed using the automated Becton-Dickinson Phoenix 100 system (Franklin Lakes, NJ, USA) for microorganisms’ biochemical identification. Next, all *S. aureus* isolates were tested for methicillin resistance according to the guidelines of the European Committee on Antimicrobial Susceptibility Testing (EUCAST, 2021) [50], using the disk diffusion method with cefoxitin antibiotic (30 µg). The resulting zones of growth inhibition were interpreted according to the breakpoint tables (EUCAST, 2021) [72] in which zone diameter ≥22 mm indicates a strain’s susceptibility to methicillin, while zone diameter <22 mm indicates the strain’s resistance to methicillin.

### 4.2. Rotating Magnetic Field Generator 

**Figure 10:**
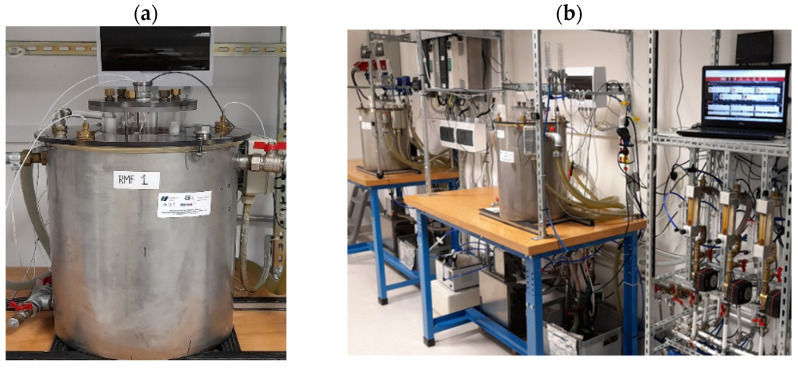
(**a**) RMF generator with (**b**) monitoring and control equipment.

The base of each RMF reactor (Figure 10) was a 3-phase, four-pole stator with an internal core diameter of 16 cm and height of 20 cm equipped with twelve groups of three coils sets [73]. The alternating current (AC) frequency supplied to the RMF generator was controlled using a Unidrive M200 inverter (Control Techniques, Nidec Industrial Automation, Poznan, Poland). The temperature in the RMF reactor chamber was maintained using a semiautomatic water fed cooling/heating system monitored by a set of temperature probes with sampling deviation in the accuracy range ±1.0 °C. The correct temperature distribution in the RMF reactor chamber was ensured by air flow supplied continuously throughout the exposure (2 L/min, 35 °C, RH 60%). The characteristics of the RMF, including the distribution of magnetic induction (*B*) in the reactor chamber were performed at an initial voltage of 100 VAC and AC frequencies of 5, 10, 25 and 50 Hz using Ansys Maxwell simulation software ver.19.1 (ANSYS, Inc., Canonsburg, PA, USA) and confirmed empirically using a teslameter (SMS-102, Asonik, Tuczno, Poland) equipped with a transverse probe.

### 4.3. Detection of mecA Gene 

Total DNA was extracted from bacterial cultures using a DNA Bacterial & Yeast Genomic Purification Kit (EURx, Gdansk, Poland), according to the manufacturer’s instructions. The presence of the *mecA* gene was detected by the PCR method using primers previously described by Oliveira and de Lencastre (2002) [74]. The PCR reaction mixture (25 µL) consisted of 6.25 µL 2x PCR Mix Plus (A&A Biotechnology, Gdansk, Poland), 0.4 µM of each primer, and 20–50 ng of DNA. The PCR conditions were as follows: initial denaturation of DNA at 95 °C for 2 min, 30 cycles (95 °C for 30 s, 55 °C for 30 s, 72 °C for 1 min) and final extension at 72 °C for 5 min [75]. PCR products were analyzed using 2% agarose gel (Prona Agarose, Burgos, Spain) electrophoresis in 1x Tris-borate-EDTA buffer (Thermo Scientific, Vilnius, Lithuania). The DNA was stained with ethidium bromide (Merck, Darmstadt, Germany), visualized under UV light and analyzed using GeneTools software (Syngene, Cambridge, UK). *S. aureus* control strains included ATCC 33591 (*mecA* positive) [76] and ATCC 6538 (*mecA* negative) [77]. PCR for non-template controls was also performed in each analysis to exclude potential DNA contamination.

### 4.4. Analysis of the Impact of RMF on Changes in Antibiotic Susceptibility

#### 4.4.1. Disc Diffusion Method

**Figure 11:**
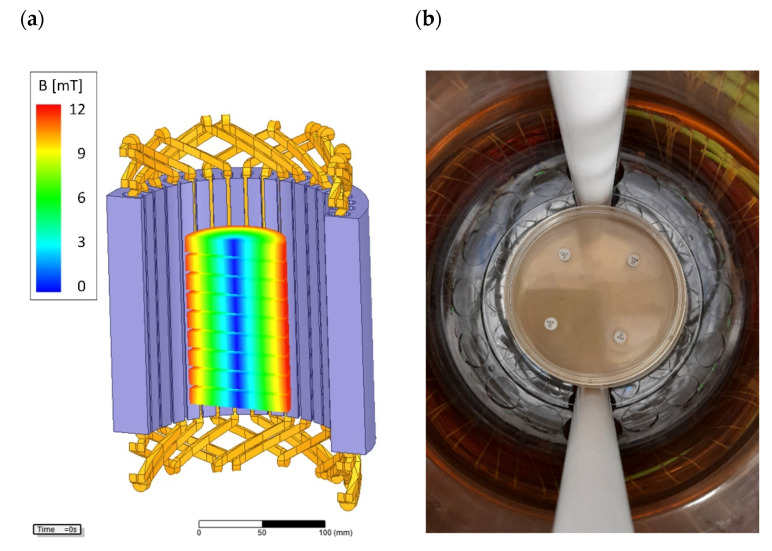
(**a**) Arrangement and location of the Petri dishes and (**b**) antibiotic discs in RMF reactor chamber.

In the first stage of the study, the impact of continuous exposure to the RMF generated at specific AC frequencies (5, 10, 25 and 50 Hz) for 18 h on the change in susceptibility to β-lactam antibiotics, including cefoxitin, was analyzed. The location of the Petri dishes in the RMF reactor chamber and the location of antibiotic discs in Petri dishes is presented in Figure 11.

The rationale behind the use of cefoxitin is the recommendation of this antibiotic by EUCAST (2021) [72] as the indicator of methicillin resistance, which, in turn, is interpreted as resistance to a prevailing majority of β-lactam antibiotics. To demonstrate whether the observed changes and analyzed dependencies in susceptibility may be applied to cephalosporins other than cefoxitin, cefepime, cefuroxime and ceftriaxone were also selected and analyzed in the same way as it was performed for cefoxitin. 

In the second stage of the study, the efficient exposure time to the RMF (generated at optimal AC frequency, which was defined on the basis of the results of the first stage), ranging from 1 to 12 h, was determined. After completion of the exposure time, the plates containing staphylococcal cultures with antibiotic discs were taken out from the RMF generator and incubated at 35 ± 1 °C without RMF until 18 h of incubation were completed (total amount of time consisting of exposure and nonexposure period). The zones of growth inhibition were measured after incubation. If inhibition zones were visible after the end of exposure to RMF, they were measured at this time point and once more after completion of entire incubation time. 

In the third stage of the study, the impact of RMF on the changes in susceptibility of different groups of β-lactams, including penicillins, cephalosporins (1st–5th generation) and carbapenems was analyzed. 

In the fourth stage of the research, the impact of the RMF on the changes in the susceptibility to cefoxitin was examined with the use of 21 MRSA strains. The rationale behind this approach was to exclude the possibility that the observed effect occurred only in three MRSA strains applied in the previous experimental stages. 

In the fifth stage, five MSSA clinical isolates and one MSSA reference strain (ATCC 6538) were exposed to the RMF in the presence of a cefoxitin-saturated disc. The aim of this experimental line was to discriminate whether the earlier observed changes in β-lactam susceptibility in the presence of RMF were related to the interaction of the RMF and antibiotics or were related to the mechanism of resistance to β-lactam antibiotics (based on the presence of the alternative PBP2a protein), which MSSA strains do not possess. 

In stages from three to five, the optimal RMF frequency (the frequency causing the greatest and stable changes in the antibiotic susceptibility of the analyzed MRSA strains) and the optimal exposure time (exposure time after which the maximal and lasting zones of growth inhibition were observed) were applied using data obtained in the previous stages of the research.

Antibiotic susceptibility testing was carried out according to the guidelines of EUCAST (2021) [50]. The bacterial cultures were adjusted to a 0.5 McFarland standard, which approximately corresponds to 1–2 × 10^8^ CFU/mL. A sterile cotton swab was used to spread inoculums evenly over the surface of the M-H agar (Graso Biotech, Jablowo, Poland) plates. After the application of antimicrobial discs on the agar surface, the bacteria were exposed to RMF in accordance with the aforementioned variants. The same bacterial cultures, incubated under the same conditions but without exposure to the RMF, were used as a control setting. Both in the RMF generator and the incubator, the same temperature (35 ± 1 °C) and relative humidity RH (60%) were maintained throughout the entire experiment.

The following antibiotic discs were used: cefoxitin (30 μg/disc), amoxicillin (10 µg/disc), ceftazidime (10 μg/disc), cephradine (30 μg/disc), cephalexin (30 μg/disc), ceftaroline (5 μg/disc), cefepime (30 μg/disc), cefuroxime (30 μg/disc), ceftraxone (30 μg/disc), cefotetan (30 μg/disc), meropenem (10 μg/disc), imipenem (10 μg/disc), ertapenem (10 μg/disc), doripenem (10 μg/disc), cefazolin (30 µg/disc) (Oxoid, Basingstoke, UK).

#### 4.4.2. Gradient MIC Strips (E-Test)

Because the disk diffusion method allows to analysis of the impact of a single concentration of an antibiotic/disk, in this experiment E-test strips with exponentially decreasing antibiotic gradient were applied. In this investigation line, the antibiotics were selected whose application, coupled with exposure to RMF, caused the largest and the smallest differences in the zones of growth inhibition in the disc diffusion method (compared to unexposed controls). The E-tests containing cefoxitin, amoxicillin, imipenem, ceftriaxone, cefuroxime, ceftazidime and cefepime were obtained from Liofilchem (Roseto degli Abruzzi, Italy), whereas the E-test containing meropenem was purchased from BioMérieux (Craponne, France).

Similarly, as in the case of the disc diffusion method, the optimal RMF frequency and exposure time, as well as unexposed controls were used. The analyses were performed on M-H agar in accordance with the E-test manufacturer’s recommendations.

### 4.5. Analysis of the Impact of RMF on Changes in the Number of Culturable Bacteria

The conducted studies also included assessment of the influence of the RMF on the number of bacterial cells expressing an ability to form colonies on the M-H agar. For this purpose, bacterial cultures prepared as for antibiotic susceptibility testing were exposed to the RMF generated at the optimal frequency and exposure time. Next, 2 mL of sterile PBS was poured onto each culture plate, mixed thoroughly with a spreader, and transferred into a sterile test tube. The process was repeated three times to remove bacteria from the agar plate. In the next step, decimal dilutions of the obtained suspension were made, inoculated on BHI (Graso Biotech, Jablowo, Poland) agar and incubated for 24 h at 37 °C. After incubation, the colonies were counted and expressed as number of CFU/mL.

### 4.6. Analysis of the Impact of RMF on Changes in the Relative Number of Live and Dead Bacterial Cells Using Fluorescence Microscopy and Picture Processing

The microbial cultures were prepared and exposed to the RMF at the optimal frequency and exposure time as performed in the analyses of the number of culturable bacteria. Staphylococcal liquid cultures were centrifuged, and the obtained cell-free supernatant was removed and replaced with 1 mL of LIVE/DEAD™ BacLight™ Bacterial Viability Kit (Invitrogen, Thermo Fisher Scientific, Bend, OR, USA) solution. The samples were then vortex-mixed, spin-centrifuged and incubated at room temperature for 15 min. After incubation, the samples were again centrifuged, and cell-free LIVE/DEAD (Live, *L*-SYTO9 dye; Dead, *D*-propidium iodine dye) solution was removed. The dyed staphylococcal pellet was resuspended in 100 µL of sterile water and introduced to the wells of a 24-well plate (VWR, Randor, PA, USA). The samples were left to dry at room temperature in darkness while shaking at 100 rpm (Lab Companion IST 30-075, Oxfordshire, UK). The rationale behind this stage was even propagation of bacterial cells throughout the surface of the plate’s well. Subsequently, the pictures of dyed staphylococcal cells were captured using Lumascope 620 (Etaluma, Carlsband, CA, USA) with 20× magnification objective Olympus IPC phase (Shinjuku, Japan). The field of vision recorded was 0.49 mm and frame size was 1200 × 1200 pixels. The excitation/emission wavelengths for SYTO9 and propidium iodide were 480/500 nm and 490/635 nm, respectively. Next, the pictures were processed using ImageJ (National Institutes of Health, Bethesda, MD, USA) software. The whole captured picture was treated as the Region of Interest (ROI). The mean grey value (MGV) of each ROI was recorded for green and red fluorescence channels and served as an estimator of changes in the number of live and dead cells, respectively. The MGV was considered the sum of gray values of all the pixels in the selection divided by the number of pixels. For RGB images recorded for the purpose of this analysis, the MGV was calculated by converting each pixel to grayscale using the Equation (1) [78]:

gray = 0.299red + 0.587green + 0.114blue(1)

In total, 24 ROIs from each channel were recorded for each sample. 

### 4.7. Analysis of the Impact of RMF on the Diffusion of Antibiotics in the Agar Medium

To analyze the potential influence of the RMF on the changes in antibiotic diffusion, the same discs as those used for the analysis of antibiotic resistance were placed on Petri dishes with 1.7% agar (agar concentration was equal to the concentration of M-H agar applied in previous experiments), and exposed to the RMF generated at 5 and 50 Hz for 30 and 120 min. After each time-point, one plate was removed from the RMF generator and, using a cork borer, cylindrical agar samples 6 mm in diameter were cut out (four samples from the proximal zone (zone 1), and eight samples from the distal zone (zone 2), representing 50% of the total agar volume in each zone). The agar samples were cut out radially starting from the edge of the antibiotic disc towards the edge of the Petri dish (Figure S7). To extract the antibiotic, the agar samples were placed in 0.5 mL of methanol (Stanlab, Lublin, Poland) in deionized water (1:1) and incubated in a shaker (250 rpm; Biosan, Riga, Latvia) at room temperature for 2 h. Then, the samples were removed, the methanol-water mixtures with the extracted antibiotics were filtered through a syringe filter (0.22 µm pore diameter) and analyzed by the liquid chromatography, tandem mass spectrometry (LC-MS/MS) technique (1260 Infinity II Series Liquid Chromatograph, Agilent, Santa Clara, CA, USA). An InfinityLab Poroshell 120 EC-C18 column (Agilent, Santa Clara, CA, USA) with a particle diameter of 2.7 µm equipped with a guard column was used for the chromatographic separation. The mass spectrometer (Ultivo G6465B, Agilent, Santa Clara, CA, USA) coupled to the chromatograph was used to detect and identify the tested analytes. Quantitative analysis was performed based on calibration curves prepared with the use of high purity antibiotic standards (Millipore Sigma, St. Louis, MO, USA). The results were converted and presented as the total concentration of antibiotic released to proximal zone 1 and distal zone 2.

### 4.8. Analysis of the Impact of RMF on Staphylococcal Cell Morphology

To assess the influence of the RMF on bacterial cell morphology, scanning electron microscopy (SEM) and transmission electron microscopy (TEM) analyses were performed. First, the cultures were prepared and exposed to the RMF as described for analyses of the number of culturable bacteria (no antibiotics were used). After exposure, cylindrical samples with a diameter of 6 mm were cut out from the M-H agar medium with the bacterial culture using a cork borer. Four samples, at least 2 cm apart, were cut out from each culture. In the second part of the experiment, the samples were prepared during the disc diffusion-based tests related to the changes in antibiotic susceptibility to cefoxitin. For this purpose, in the control setting (RMF-unexposed cultures with no inhibition zones), the agar samples were cut out next to the antibiotic discs and, in the case of the RMF-exposed samples, from the edge of the inhibition zones.

#### 4.8.1. Scanning Electron Microscopy

All collected samples were washed gently with PBS and fixed by immersion in 3% glutaraldehyde (POCH, Gliwice, Poland) for 15 min at room temperature. The samples were rinsed twice with PBS to remove the fixative. Dehydration in increasing concentrations of ethanol (25, 50, 60, 70, 80, 90, and 100% POCH) was performed for 10 min per solution. The ethanol was then rinsed off, and the samples were dried at room temperature. Next, the samples were covered with gold and palladium (60:40; sputter current, 40 mA; sputter time, 50 s) using a Quorum machine (Quorum International, Fort Worth, TX, USA) and examined under a Zeiss EVO MA25 scanning electron microscope (SEM) (Carl Zeiss, Jena, Germany). 

#### 4.8.2. Transmission Electron Microscopy

The samples were fixed in 2% glutaraldehyde (POCH) and centrifuged (5 min, 50 µf). Contrasting was performed with 2% uranyl acetate (MicroShop, Piaseczno, Poland) (8 h) and 2% osmium tetroxide (Agar Scientific, Stansted, UK) (2 h) in the dark. The material was then passed through an ascending alcohol series (POCH from 30% to 99.8%) and embedded in epoxy resin (Agar Scientific, Stansted, UK). Sections of 60 nm thickness were prepared from the resin blocks using an UltraMicrotome Leica EMUC7 (Leica, Wetzlar, Germany) and placed on copper grids (400 Mesh) with formvar film and carbon coating (Agar Scientific, Stansted, UK). Imaging was performed using a JEOL 1200, (JEOL, Tokyo, Japan) microscope.

### 4.9. Analysis of the RMF Post-Exposure Effect

This experiment aimed to assess whether the changes in antibiotic susceptibility occur only during the RMF exposure, or whether they remain fixed as a result of the exposure performed prior to the antibiotic testing. Liquid bacterial cultures of cell density equal to 0.5 of McFarland turbidity standard prepared in a M-H medium were exposed to RMF at the optimal frequency and exposure time. Next, the cultures were centrifuged, the pellet resuspended in PBS to obtain the initial cell density and used as an inoculum for the antibiotic (cefoxitin, cefuroxime, cefepime and ceftriaxone) susceptibility test. Growth inhibition zones were compared with the zones obtained in the control cultures performed using the inoculum never exposed to the RMF.

### 4.10. Statistical Analysis

The data obtained in this study (number of culturable bacteria, mean grey value and concentrations of antibiotics in agar in the control and RMF-exposed settings) were presented as means ± standard errors of the means (SEM) obtained from at least three different measurements (plus technical repetitions). Statistical differences between RMF-exposed and control, unexposed settings were determined by one-way analysis of variance (ANOVA) and Tukey’s post hoc test. Differences were considered significant at a level of *p* < 0.05. The statistical analyses were conducted using Statistica 12.5 (StatSoft, Inc. Tulsa, OK, USA).

## Figures and Tables

**Table 1:** Growth inhibition zones (mm) of MRSA strains around discs with β-lactam antibiotics depending on RMF frequency (Hz).

Strain		RMF Frequency			
	C	5 Hz	10 Hz	25 Hz	50 Hz
	Cefoxitin				
ATTC 33591	12	18	15	16	15
MRSA 1	6	16	14	16	14
MRSA 2	7	21	15	17	15
	Cefepime				
ATTC 33591	10	16	15	16	14
MRSA 1	6	19	14	18	13
MRSA 2	6	18	6 (14p.)	6 (16p.)	6
	Cefuroxime				
ATTC 33591	6	22	20	20	18
MRSA 1	6	23	19	21	18
MRSA 2	6	15	6 (14p.)	14	6
	Ceftriaxone				
ATTC 33591	6	18	15	17	16
MRSA 1	6	15	14	15	13
MRSA 2	6	15	6 (13p.)	15	6

Differences in the diameter of the growth inhibition zones between three repetitions of the experiment did not exceed ±1 mm. C—unexposed control culture; “p”—partial growth of bacteria around the antibiotic discs.

**Table 2:** Growth inhibition zones (mm) of MRSA strains around discs with β-lactam antibiotics depending on the RMF exposure (5 Hz) duration.

	Strain																							
	ATCC 33591								MRSA 1								MRSA 2							
	FOX		FEP		CXM		CRO		FOX		FEP		CXM		CRO		FOX		FEP		CXM		CRO	
*t*	C	RMF	C	RMF	C	RMF	C	RMF	C	RMF	C	RMF	C	RMF	C	RMF	C	RMF	C	RMF	C	RMF	C	RMF
1	13	13	10	10	6	6	6	6	6	6	6	6	6	6	6	6	7	7	6	6	6	6	6	6
2	12	13	9	11	6	6	6	10	6	6	6	6	6	6	6	6	6	10	6	6	6	6	6	6
3	12	13	9	12	6	16	6	12	6	11	6	6	6	6	6	6	7	12	6	6	6	6	6	6
4	12	14	10	12	6	15	6	12	6	14	6	14	6	19	6	14	7	13	6	13	6	14	6	6
5	12	15	9	13	6	16	6	13	6	15	6	13	6	20	6	15	7	14	6	15	6	15	6	13
6	12	15	9	13	6	17	6	13	6	15	6	15	6	19	6	15	7	14	6	17	6	15	6	13
7	13	16	10	14	6	17	6	13	6	16	6	15	6	21	6	15	7	13	6	17	6	15	6	13
8	12	16	10	15	6	18	6	13	6	16	6	15	6	20	6	15	7	14	6	16	6	15	6	12
9	12	16	10	14	6	18	6	15	6	16	6	16	6	20	6	15	7	16	6	18	6	15	6	13
10	12	17	10	15	6	19	6	16	6	16	6	17	6	21	6	15	7	18	6	18	6	15	6	13
11	12	18	10	16	6	20	6	17	6	16	6	18	6	22	6	15	7	20	6	18	6	15	6	14
12	12	18	10	16	6	22	6	18	6	16	6	19	6	23	6	15	7	21	6	18	6	15	6	15
18 *	12	18	10	16	6	22	6	18	6	16	6	19	6	23	6	15	7	21	6	18	6	15	6	15

Cultures with antibiotic discs were exposed to the RMF for a specified time (*t*), ranging from 1 to 12 h, and then the plates were transferred to the incubator until the 18 h period of incubation was completed. 18 *—constant exposure to the RMF for 18 h. C—control culture unexposed to RMF. The differences in the diameter of the growth inhibition zones between three repetitions of the experiment did not exceed ±1 mm. FOX—cefoxitin, FEP—cefepime, CXM—cefuroxime, CRO—ceftriaxone.

**Table 3:** Growth inhibition zones (mm) of MRSA strains around discs with different β-lactam antibiotics in RMF-exposed (5 Hz) and control, unexposed cultures.

Strain	C	RMF	C	RMF	C	RMF	C	RMF
	Cefazolin		Cefotetan		Cefradine		Cefalexin	
ATCC 33591	11	18	9	12	16	16	10	15
MRSA 1	6	18	6	11	6	12	6	9
MRSA 2	6	12	6	14	6	6	6	6
	Ceftaroline		Meropenem		Imipenem		Ertapenem	
ATCC 33591	21	23	9	15	31	35	6	16
MRSA 1	19	22	6	18	6	22	6	15
MRSA 2	20	22	6	15	10	20	6	17
	Ceftazidime		Doripenem		Amoxicilin			
ATCC 33591	6	6	6	13	6	6		
MRSA 1	6	6	6	12	6	6		
MRSA 2	6	6	6	12	6	6		

Differences in the diameter of the growth inhibition zones between three repetitions of the experiment did not exceed ±1 mm. C—control culture unexposed to RMF.

**Table 4:** MIC values (µg/mL) of β-lactam antibiotics for MRSA strains in control and RMF-exposed (5 Hz) cultures.

	Strain					
	ATCC 33591		MRSA 1		MRSA 2	
	C	RMF	C	RMF	C	RMF
Cefoxitin	24	16	256	24	256	6
Amoxicilin	8	6	24	6	24	12
Imipenem	1.5	1	32	0.19	32	1.5
Meropenem	1	0.75	32	1.5	32	4
Ceftriaxone	32	24	256	16	256	24
Cefuroxime	12	8	256	8	256	12
Ceftazidime	32	32	256	32	256	96
Cefepime	32	16	256	16	256	64

C—control culture unexposed to RMF; there were no differences in MIC values between three separate experiments.

**Table 5:** Zones of growth inhibition (mm) of MRSA strains around cefoxitin discs in control and RMF-exposed (5 Hz) cultures.

Strain	C	RMF	Strain	C	RMF
ATCC 33591	12	18	MRSA 12	12	16
MRSA 1	6	16	MRSA 13	6	20
MRSA 2	7	21	MRSA 14	9	15
MRSA 3	6	17	MRSA 15	12	18
MRSA 4	6	18	MRSA 16	6	14
MRSA 5	6	16	MRSA 17	7	18
MRSA 6	6	16	MRSA 18	11	16
MRSA 7	6	21	MRSA 19	6	14
MRSA 8	12	16	MRSA 20	6	16
MRSA 9	6	13	MRSA 21	15	19
MRSA 10	6	19	MRSA 22	6	14
MRSA 11	12	19	MRSA 23	6	12

Differences in the diameter of the growth inhibition zones between three repetitions of the experiment did not exceed ±1 mm. C—control culture unexposed to RMF.

**Table 6:** MIC values (µg/mL) for β-lactam antibiotics in control and RMF-exposed (5 Hz) MSSA cultures.

Strain	Cefoxitin		Cefepime		Cefuroxime		Ceftriaxone	
	C	RMF	C	RMF	C	RMF	C	RMF
ATCC 6538	3	3	3	2	1.5	1	3	3
MSSA 1	3	3	4	3	1	0.75	2	2
MSSA 2	3	3	4	3	2	1	6	3
MSSA 3	3	3	4	3	1.5	1	6	4
MSSA 4	3	3	3	2	1	0.75	3	2
MSSA 5	3	3	8	4	2	1.5	8	6

C—control culture unexposed to RMF. There were no differences in MIC values between three separate experiments.

## Data Availability

The data presented in this study are available on request from the corresponding author.

## Supplementary Materials

The following are available online at https://www.mdpi.com/article/10.3390/ijms222212397/s1.
