# Supplementary material for: Rotating Magnetic Field Increases β-Lactam Antibiotic Susceptibility of Methicillin-Resistant Staphylococcus aureus Strains
Source: Int J Mol Sci. 2021 Nov 17;22(22):12397. doi: 10.3390/ijms222212397 (PMC8618647; doi:10.3390/ijms222212397)
Supplement: Supplementary file 1 [file ijms-22-12397-s001.zip › ijms-1414525-supplementary.pdf]

Supplementary material

Rotating Magnetic Field increases β-lactam antibiotic susceptibility of methicillin-resistant *Staphylococcus aureus* strains

Marta Woroszyło[a], Daria Ciecholewska-Juśko[a], Adam Junka[b,c*], Radosław Drozd[a], Marcin Wardach[d], Paweł Migdał[e], Patrycja Szymczyk-Ziółkowska[f], Daniel Styburski[g], Karol Fijałkowski[a*]

[a] Department of Microbiology and Biotechnology, Faculty of Biotechnology and Animal Husbandry, West Pomeranian University of Technology in Szczecin, Piastów 45, 70-311 Szczecin, Poland; marta.woroszylo@zut.edu.pl; daria.ciecholewska@zut.edu.pl; radoslaw.drozd@zut.edu.pl; karol.fijalkowski@zut.edu.pl

[b] Department of Pharmaceutical Microbiology and Parasitology, Faculty of Pharmacy, Medical University of Wroclaw, Borowska 211a, 50-534 Wrocław, Poland; adam.junka@umed.wroc.pl

[c] Laboratory of Microbiology, Łukasiewicz Research Network–PORT Polish Center for Technology Development, 54-066 Wrocław, Poland; adam.junka@port.lukasiewicz.gov.pl

[d] Faculty of Electrical Engineering, West Pomeranian University of Technology in Szczecin, Sikorskiego 37, 70-313 Szczecin, Poland; marcin.wardach@zut.edu.pl

[e] Department of Environment, Hygiene and Animal Welfare, Faculty of Biology and Animal Science, Wroclaw University of Environmental and Life Sciences, Chełmońskiego 38C, 51-630 Wrocław, Poland; pawel.migdal@upwr.edu.pl

[f] Centre for Advanced Manufacturing Technologies (CAMT/FPC), Faculty of Mechanical Engineering, Wroclaw University of Science and Technology, Łukasiewicza 5, 50-371, Wrocław, Poland; patrycja.e.szymczyk@pwr.edu.pl

[g] Laboratory of Chromatography and Mass Spectroscopy, Faculty of Biotechnology and Animal Husbandry, West Pomeranian University of Technology in Szczecin, Klemensa Janickiego 29, 71-270, Szczecin, Poland; daniel.styburski@zut.edu.pl

*Correspondence:
KF: karol.fijalkowski@zut.edu.pl; + 48 91-449-6714;
AJ: adam.junka@umed.wroc.pl; +48 88-922-9341.

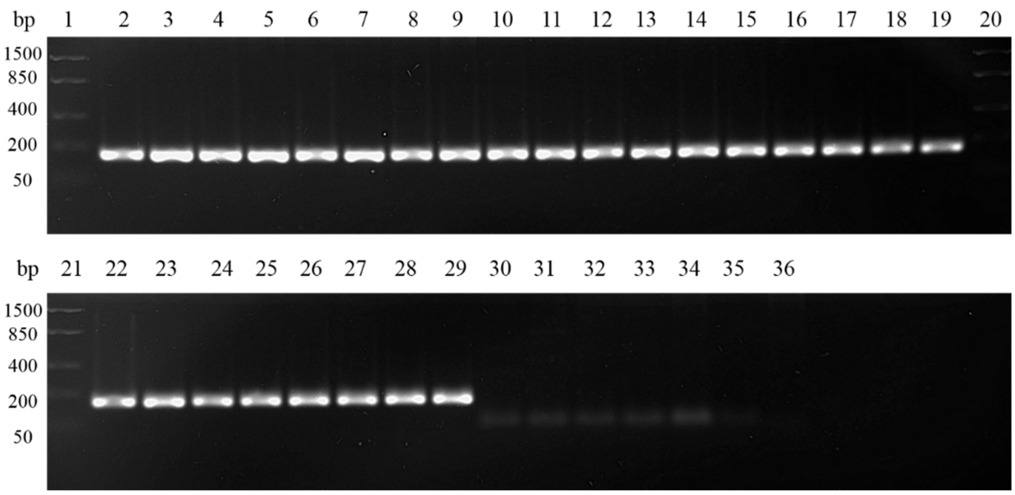

Figure S1. Detection of *mecA* gene.
1, 20, 21 - FastRuler DNA Ladder; 2, 19, 22 - *S. aureus* ATCC 33591 (*mecA* positive control); 3-18 - MRSA; 23-29 - MRSA; 30-34 - MSSA; 35 - no template control; 36 - *S. aureus* ATCC 6538 (*mecA* negative control).

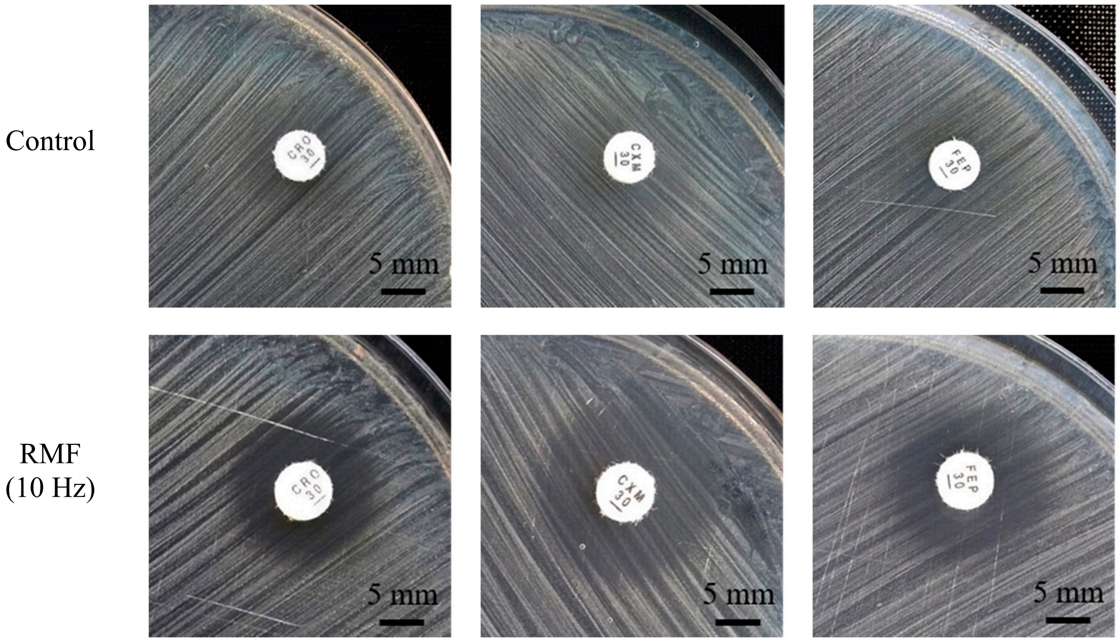

Figure S2. Partial growth around the antibiotic discs of MRSA 2 strain exposed to the RMF (10 Hz).
CRO - ceftriaxone, CXM - cefuroxime, FEP - cefepime.

Table S1. Growth inhibition zones (mm) of MRSA strains measured after exposure to the RMF (5 Hz) and after further (to 18th hour) incubation w/o RMF (5 Hz/Inc).

	Strain																							
	ATCC 33591								MRSA 1								MRSA 2							
t	FOX		FEP		CXM		CRO		FOX		FEP		CXM		CRO		FOX		FEP		CXM		CRO	
	5 Hz	5 Hz/Inc	5 Hz	5 Hz/Inc	5 Hz	5 Hz/Inc	5 Hz	5 Hz/Inc	5 Hz	5 Hz/Inc	5 Hz	5 Hz/Inc	5 Hz	5 Hz/Inc	5 Hz	5 Hz/Inc	5 Hz	5 Hz/Inc	5 Hz	5 Hz/Inc	5 Hz	5 Hz/Inc	5 Hz	5 Hz/Inc
7	17	16	15	14	18	17	14	13	17	16	16	15	21	21	16	15	14	13	17	17	16	15	13	13
8	17	16	15	15	18	18	14	13	17	16	16	15	21	20	16	15	15	14	17	16	15	15	13	12
9	17	16	15	14	18	18	16	15	16	16	16	16	21	20	15	15	16	16	18	18	15	15	13	13
10	18	17	15	15	19	19	17	16	16	16	17	17	21	21	15	15	18	18	18	18	15	15	13	13
11	18	18	16	16	20	20	17	17	16	16	18	18	22	22	15	15	20	20	18	18	15	15	14	14
12	18	18	16	16	22	22	18	18	16	16	19	19	23	23	15	15	21	21	18	18	15	15	15	15
18*	18	-	16	-	22	-	18	-	16	-	19	-	23	-	15	-	21	-	18	-	15	-	15	-

t - time (h) of exposure to the RMF. 18* - constant exposure to the RMF for 18 h. The differences in the diameter of the growth inhibition zones between three repetitions of the experiment did not exceed ± 1 mm. FOX - cefoxitin, FEP - cefepime, CXM - cefuroxime, CRO – ceftriaxone.

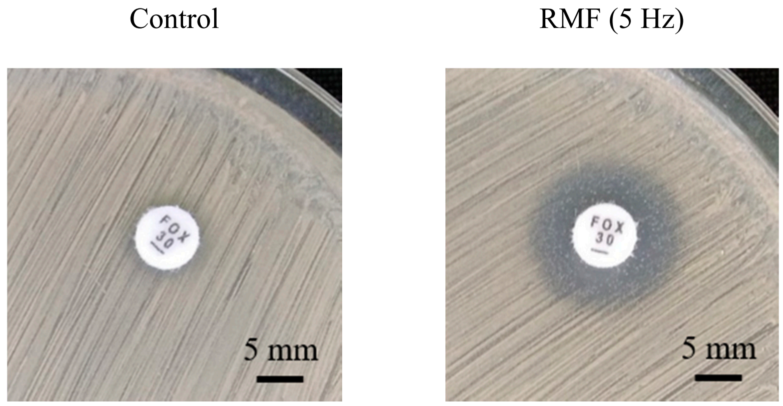

Figure S3. Partial growth around the antibiotic discs with cefoxitin (FOX) of MRSA 1 strain after exposure to the RMF (5 Hz) for 2 h.

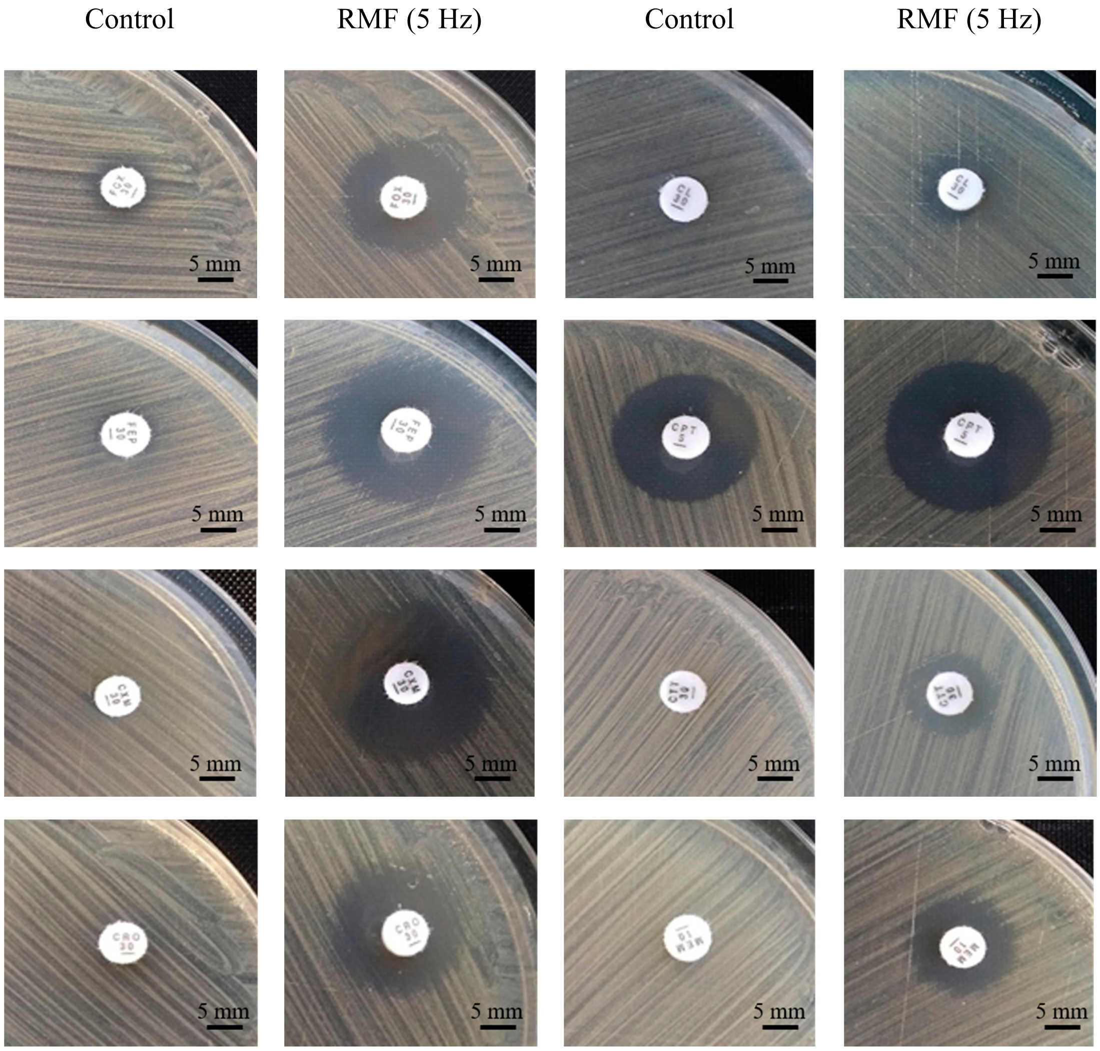

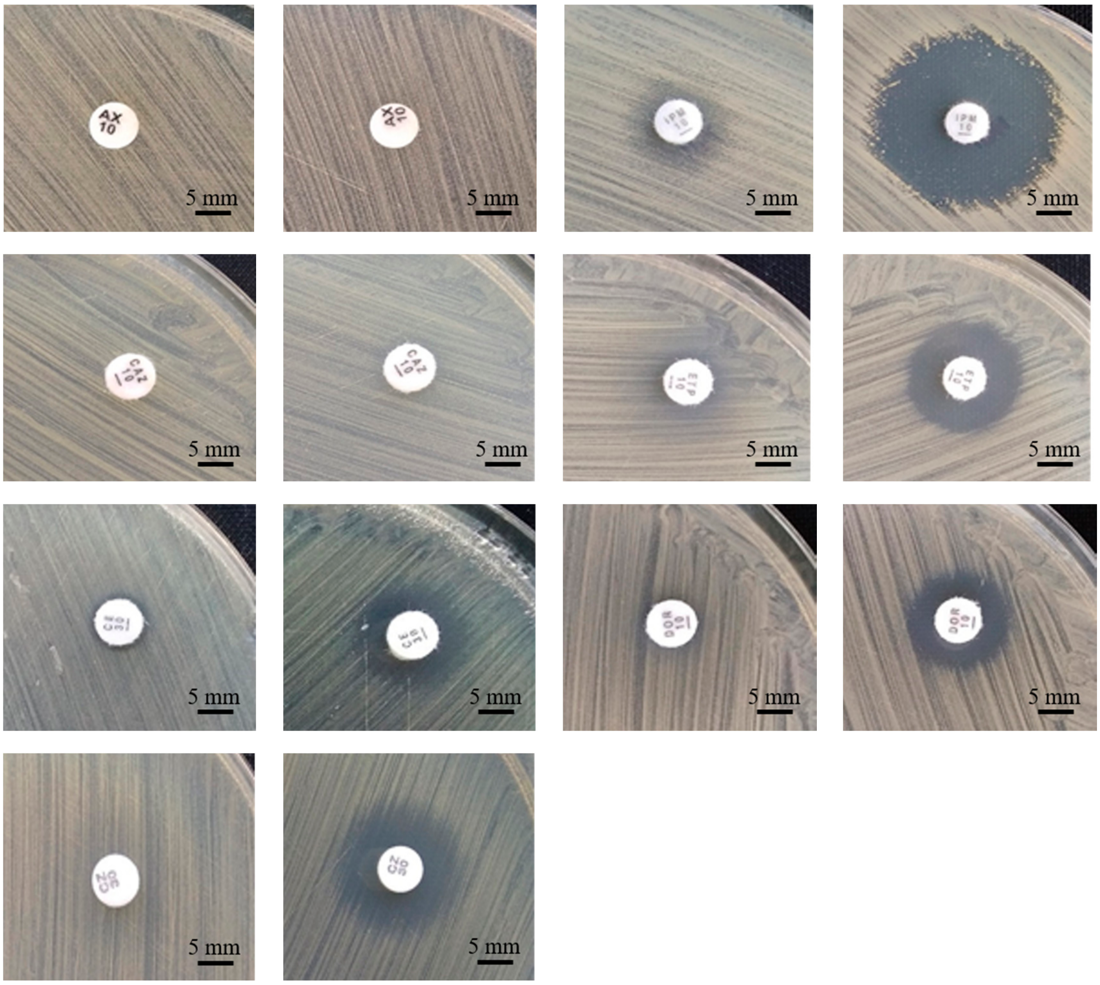

Figure S4. Representative pictures of growth inhibition zones in control and RMF-exposed (5 Hz) MRSA 1 cultures around discs with β-lactam antibiotics.
FOX - cefoxitin, FEP - cefepime, CXM - cefuroxime, CRO - ceftriaxone, AX - amoxicillin, CAZ - ceftazidime, CE - cephradine, CZ - cefazolin, CL - cephalexin, CPT - ceftaroline, CTT - cefotetan, MEM - meropenem, ETP - ertapenem, DOR - doripenem.

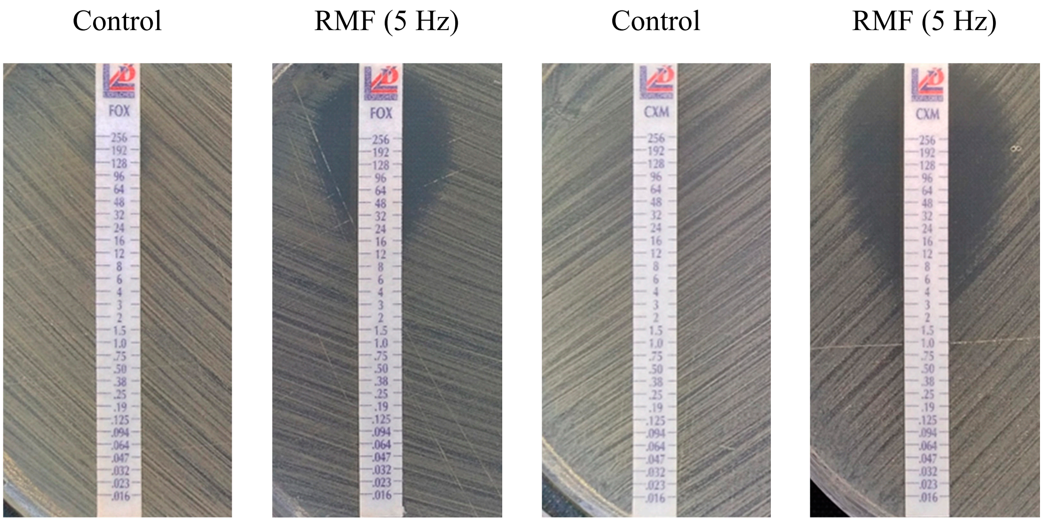

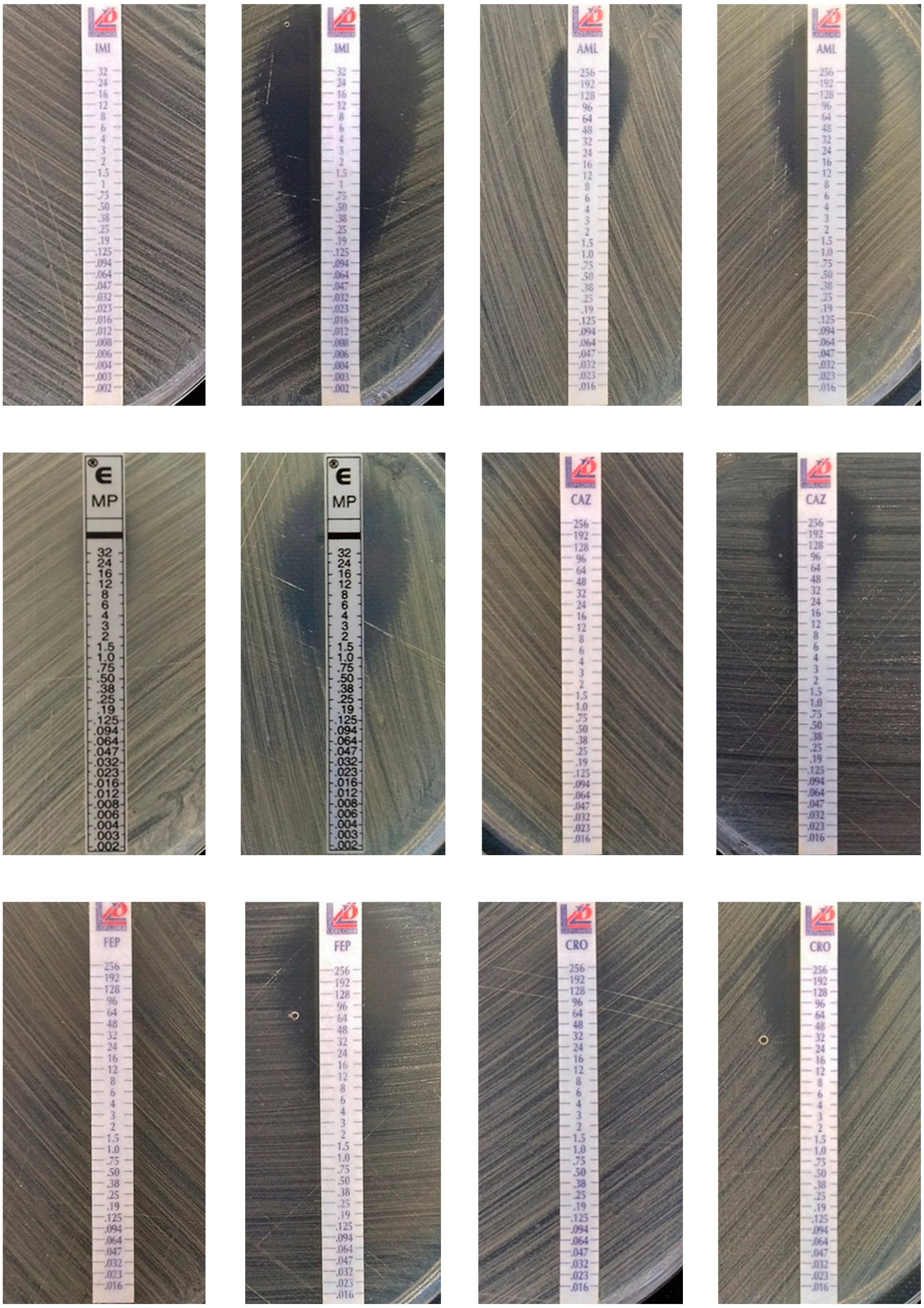

Figure S5. Representative pictures of gradient MIC strips (E-tests) with β-lactam antibiotics in control and RMF-exposed (5 Hz) cultures of the MRSA 1 strain.
FOX - cefoxitin, IMI - imipenem, MP - meropenem, FEP - cefepime, CXM - cefuroxime, AML - amoxicilin, CAZ - ceftazidime, CRO - ceftriaxone.

Table S2. Zones of growth inhibition (mm) for β-lactam antibiotics in control and RMF-exposed (5 Hz) MSSA cultures.

	**Cefoxitin**		**Cefepime**		**Cefuroxime**		**Ceftriaxone**	
	C	RMF	C	RMF	C	RMF	C	RMF
ATCC 6538	27	27	22	22	26	26	24	24
MSSA 1	29	29	26	26	34	34	29	29
MSSA 2	26	26	22	22	27	27	23	23
MSSA 3	24	24	26	26	29	29	24	24
MSSA 4	28	28	28	28	34	34	29	29
MSSA 5	28	28	26	26	34	34	27	27

The differences in the diameter of the growth inhibition zones between three repetitions of the experiment did not exceed ± 1 mm. C - control culture un-exposed to RMF.

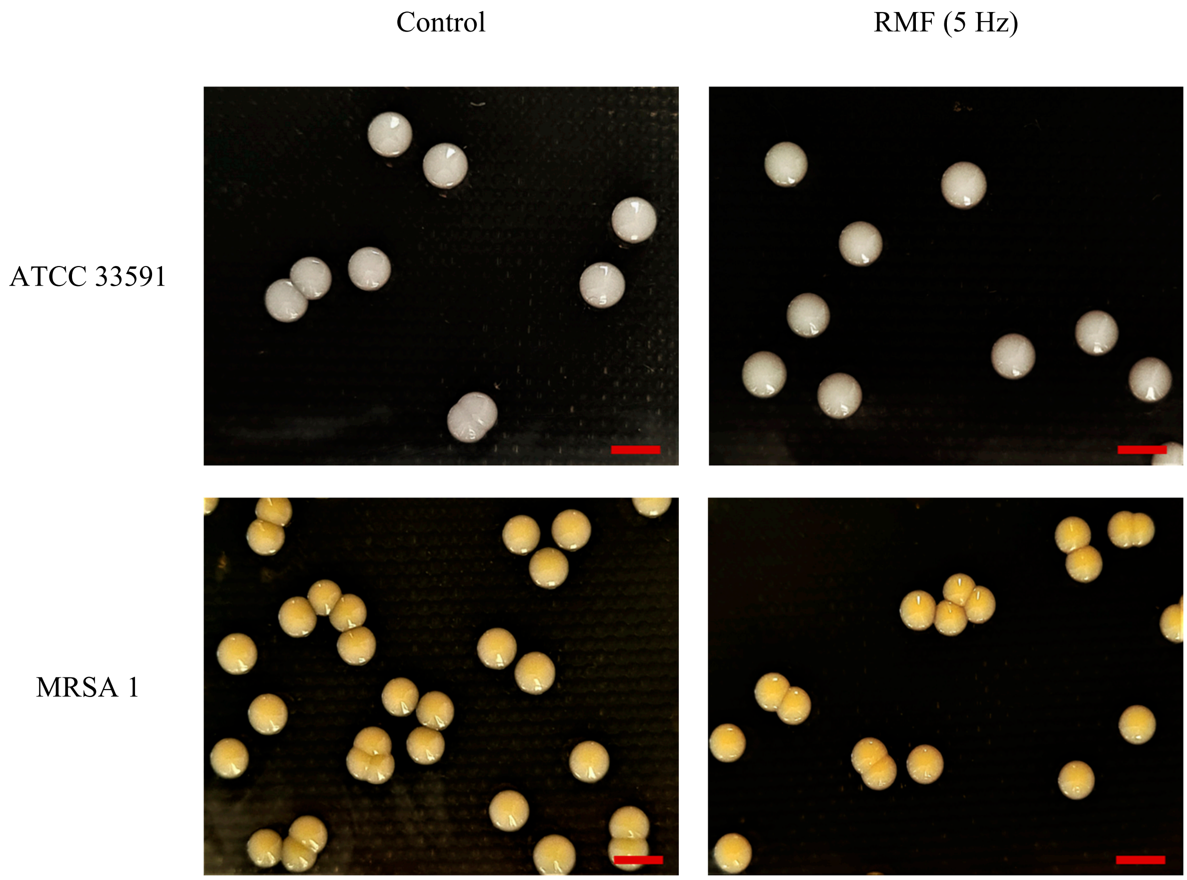

MRSA 2

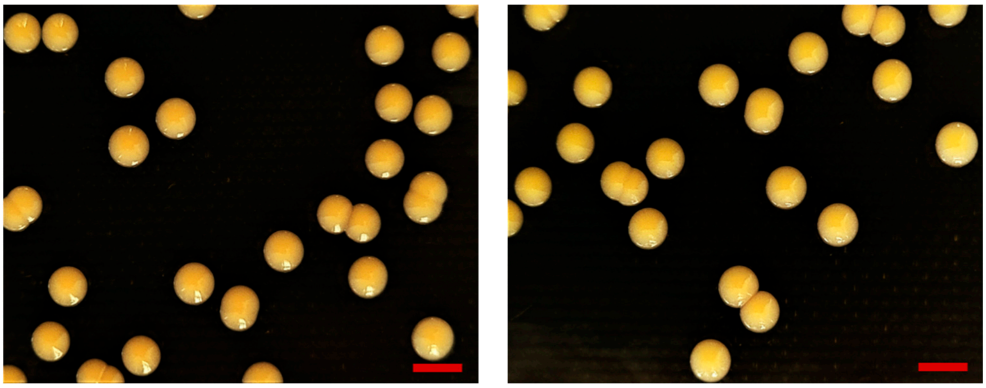

Figure S6. Morphology of the bacterial colonies of MRSA strains formed after 12 h of RMF exposure (5 Hz) in comparison to control cultures un-exposed to RMF.
Scale bars represent 2 mm.

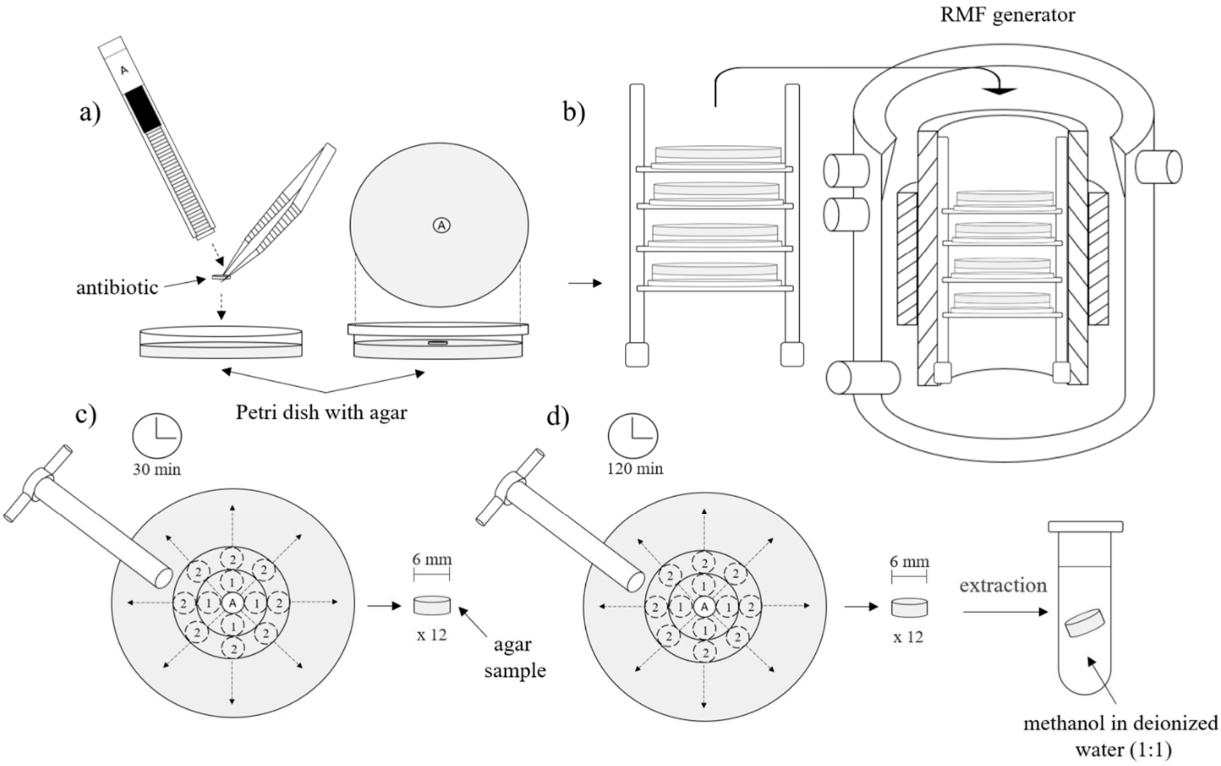

Figure S7. Schematic presentation of the analysis of the impact of RMF on the diffusion of antibiotics in the agar medium.
1 - agar disc from proximal zone (zone 1); 2 - agar disc from the distal zone (zone 2).

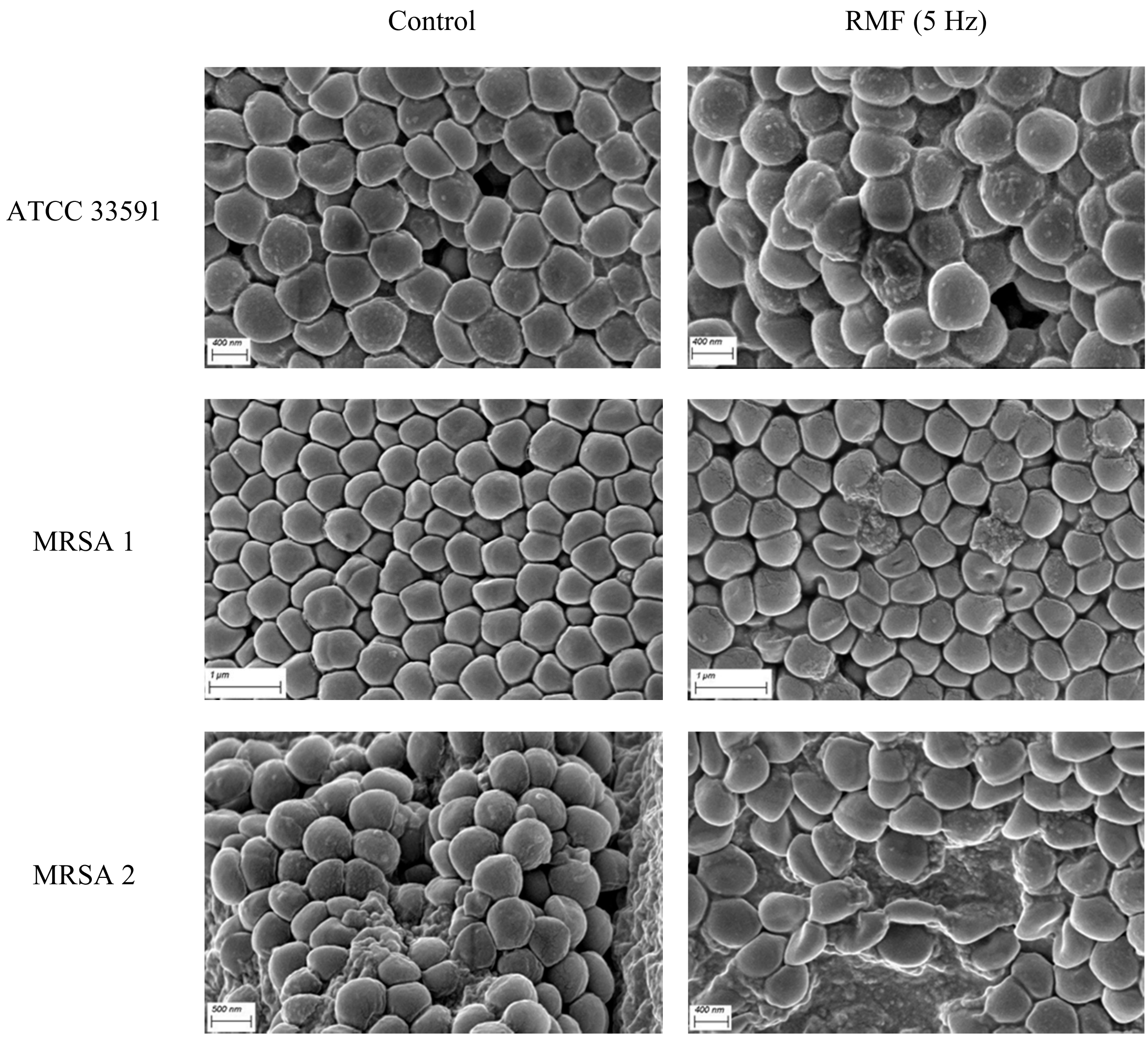

Figure S8. Scanning electron microscopy images of MRSA cells un-exposed and exposed to the RMF (5 Hz).

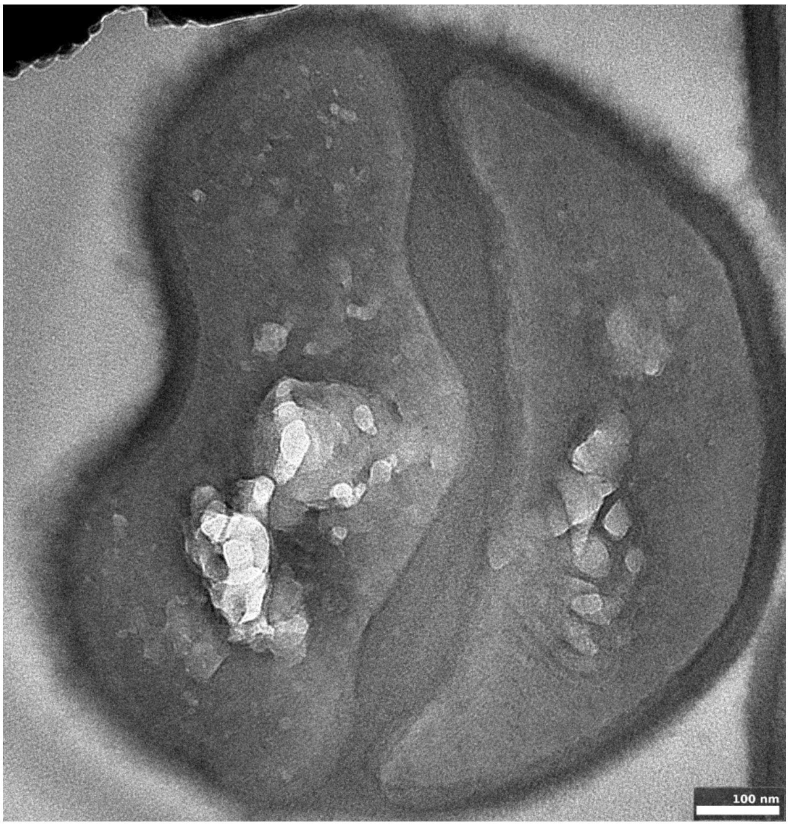

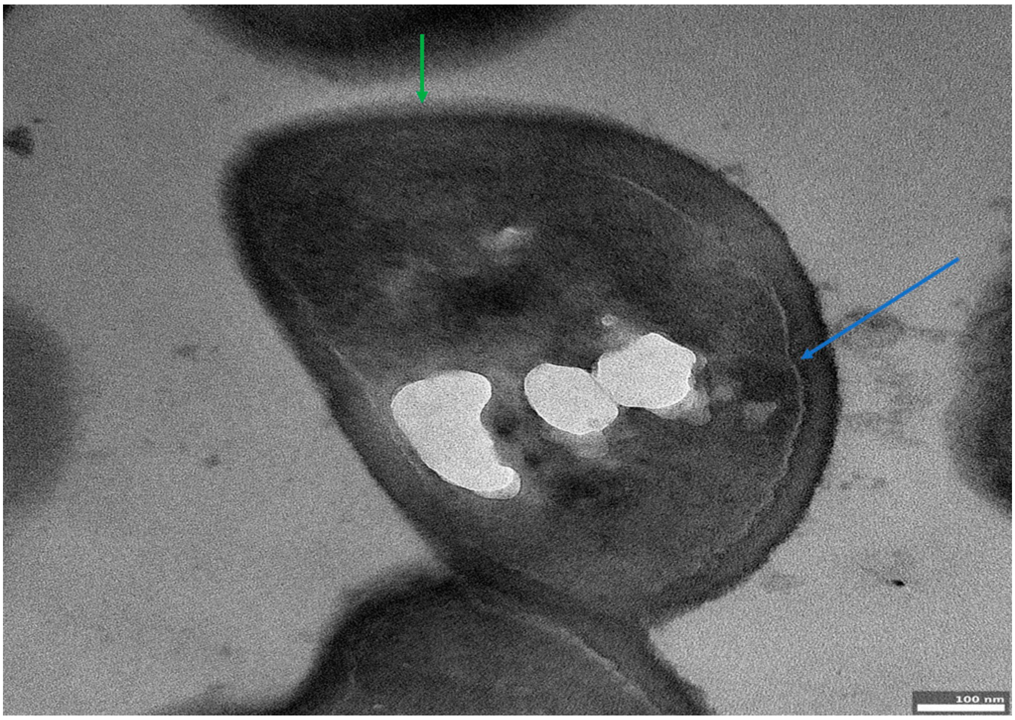

Figure S9. The magnified regions showing impact of RMF on staphylococcal cell.

Upper picture – change of cell's morphology; lower picture, blue arrow – contraction of cytoplasm; green arrow – loss of cell wall density.

Table S3. RMF post-exposure effect.

Strain	Cefoxitin		Cefuroxime	
	C	RMF	C	RMF
ATTC 33591	12	12	6	6
MRSA 1	6	6	6	6
MRSA 2	7	7	6	6
	Cefepime		Ceftriaxone	
	C	RMF	C	RMF
ATTC 33591	10	10	6	6
MRSA 1	6	6	6	6
MRSA 2	6	6	6	6

Liquid bacterial cultures of the cell density equal to 0.5 of McFarland turbidity standard prepared in a M-H medium were exposed to the RMF (5 Hz) for 12 h. Next, the cultures were centrifuged, the pellet resuspended in PBS to obtain the initial cell density and used as an inoculum for antibiotic susceptibility test. The growth inhibition zones (mm) were compared with the zones obtained in the control cultures performed using the inoculum never exposed to the RMF.
